# Spatiotemporal synergy of a coordination decomposition bifunctional strategy for ultra-stable aqueous zinc anodes

**DOI:** 10.1039/d6sc04691f

**Published:** 2026-07-03

**Authors:** Yu-Xuan Xiao, Si-Ze Wang, Yang Su, Han-Hao Liu, Zhen-Yi Gu, Xiao-Tong Wang, Jia-Lin Yang, Bin Fu, Long-Xin Zhang, Wen-Ze Luo, Jujun Yuan, Hongbo Zhang, Xinlu Wang, Jinxian Wang, Xing-Long Wu

**Affiliations:** a School of Chemistry & Environmental Engineering, Changchun University of Science and Technology Changchun Jilin 130022 P. R. China suyang@cust.edu.cn; b State Key Laboratory of Integrated Optoelectronics, MOE Key Laboratory for UV Light-Emitting Materials and Technology, Northeast Normal University Changchun China xinglong@nenu.edu.cn; c School of Intelligent Manufacturing and Future Energy, Gannan Normal University Ganzhou Jiangxi 341000 China

## Abstract

Aqueous zinc-ion batteries (AZIBs) face a critical bottleneck originating from the intrinsic interplay between solvated water reactivity and uncontrolled interfacial nucleation, which collectively trigger hydrogen evolution and dendrite formation on the Zn anode. Herein, we propose a spatiotemporal decoupling approach. Two complementary agents, namely PEGDME and FEC, are co-introduced into a conventional ZnSO_4_ electrolyte, achieving synergistic modulation across spatial and temporal scales. In the spatial dimension, the highly Lewis basic PEGDME selectively resides in the primary Zn^2+^ solvation sheath. *Via* multidentate Zn–O bonding, it expels active water species and thus markedly reduces the HER in the bulk electrolyte. Meanwhile, PEGDME assumes a flat lying orientation on the Zn(002) basal plane, accelerating lateral surface migration of Zn adatoms while inhibiting three dimensional dendritic nucleation. In the temporal domain, the electron withdrawing fluorine substituents confer FEC with a reduced LUMO level and attenuated Lewis basicity, which hinders its competition for the inner coordination sphere. Consequently, FEC preferentially adheres to surface irregularities such as step edges, and subsequently decomposes reductively to generate a ZnF_2_ rich solid electrolyte interphase (SEI). This *in situ* formed SEI continuously deactivates parasitic sites, suppresses the HER, and directs homogeneous Zn plating. Through this functional decoupling where PEGDME governs solvation regulation and surface diffusion, while FEC is responsible for defect passivation and interface stabilization, the fundamental suppression of the HER and dendrite growth is achieved. Experimental results demonstrate that the Zn//Zn symmetric battery achieves stable cycling for over 1100 hours at 1 mA cm^−2^ and 1 mA h cm^−2^, while the Zn//Cu asymmetric battery delivers an average coulombic efficiency (CE) as high as 99.2% over 200 cycles. This work presents a novel paradigm for the synergistic design of solvation engineering and interface engineering, offering an effective pathway for constructing ultra-stable aqueous zinc anodes.

## Abstract 2

We propose a coordination–decomposition strategy: PEGDME enters the solvation sheath and adsorbs on Zn(002) to promote 2D diffusion, while FEC anchors on Zn(101) to form a ZnF_2_-rich SEI, achieving dendrite-free deposition with 99.2% CE.

## Graphical abstract

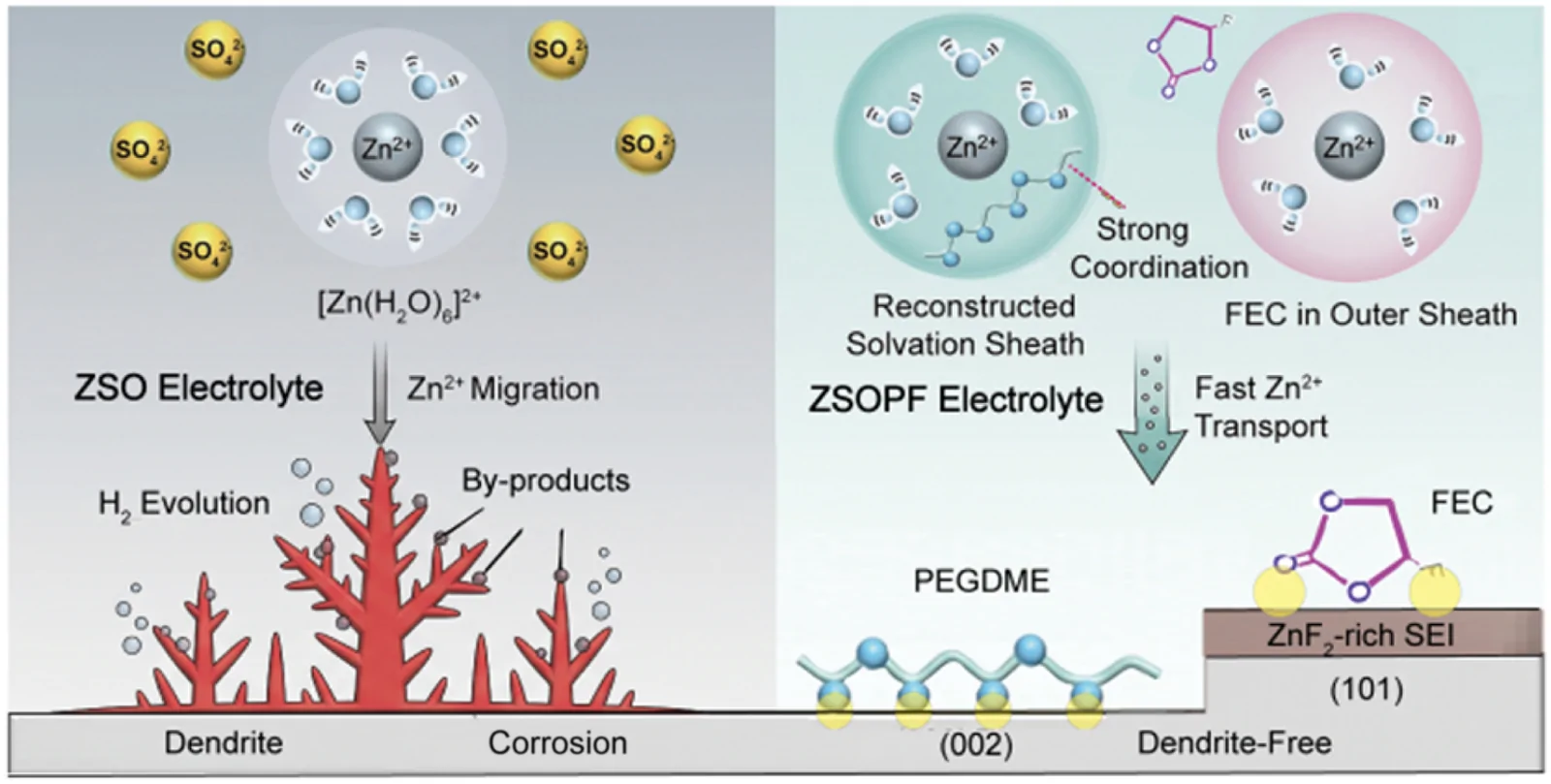

## Introduction

Grid scale energy storage has recently spurred great interest in aqueous zinc ion batteries (AZIBs) due to their multiple advantages, including intrinsic operational safety, low material cost, a high theoretical specific capacity reaching 820 mA h g^−1^, a favorable redox potential of −0.76 V (relative to the SHE), and environmental benignity.^1–3^ Unlike lithium ion systems that require flammable organic solvents and strict anhydrous assembly conditions, AZIBs rely on water based electrolytes, thereby simplifying production processes and substantially reducing safety risks.^4,5^ However, two intertwined parasitic processes at the zinc anode interface, the hydrogen evolution reaction (HER) and dendrite growth severely hinder their practical application. These issues not only reduce coulombic efficiency (CE) and shorten cycle life but also pose safety hazards during long-term operation.^6–8^

The fundamental origins of both the HER and dendritic growth lie in the unique solvation configuration of Zn^2+^ and the subsequent interfacial reactions that follow.^9,10^ In typical ZnSO_4_ containing electrolytes, Zn^2+^ ions are primarily coordinated by water molecules, forming a primary solvation shell denoted as [Zn(H_2_O)_6_]^2+^. These coordinated water species display substantial chemical reactivity and are prone to proton reduction, which continuously generates hydrogen bubbles and elevates the local pH.^11,12^ Simultaneously, the kinetics of Zn^2+^ deposition onto the anode are governed by two competing factors: surface diffusion and nucleation events. On pristine Zn surfaces, the high activation barrier for adatom movement commonly leads to three dimensional (3D) island type growth, which readily evolves into acute dendritic morphologies.^13,14^ These dendrites can penetrate the separator, cause internal shorting, and speed up electrolyte decomposition. Critically, the HER and dendrite growth are mutually reinforcing. The pH increase arising from the HER promotes surface passivation and non-uniform deposition, which in turn worsens dendrite propagation.^15–17^ Therefore, decoupling these coupled failure mechanisms is key to achieving ultra-stable zinc anodes.

Considerable research has been dedicated to overcoming these obstacles. Numerous electrolyte additives have been investigated for their ability to modify the solvation sheath. Examples include organic cosolvents like ethylene glyco^18^ and dimethyl sulfoxide,^19^ as well as ionic liquids,^20^ all designed to lower water activity and inhibit the HER. Furthermore, metal cations,^21^ organic molecules,^22^ and inorganic salts^23^ have been widely studied as potential additives. Fluoroethylene carbonate (FEC) serves as an illustrative example. It has been shown to effectively construct a ZnF_2_ rich solid electrolyte interphase (SEI) on Zn anodes, consequently restraining dendrite development and alleviating parasitic side reactions,^24^ thereby inhibiting dendrite evolution and mitigating parasitic reactions. Surface modification strategies, such as carbonaceous coatings,^25^ metal organic frameworks,^26^ and polymeric films,^27^ aim to physically block water access and guide homogeneous Zn electrodeposition. Separately, crystallographic engineering strategies have been developed to encourage Zn(002) texture formation, which benefits planar deposition behavior.^28–30^ Nevertheless, most existing approaches tackle only a single aspect of the overall problem. Additives that modulate solvation, for instance, may reduce the HER but often fail to regulate nucleation overpotential or adatom mobility, thereby leaving dendrite growth uncontrolled. Conversely, protective interfacial layers can limit dendrite formation but cannot remove the inherent water reactivity present in the bulk electrolyte. Moreover, many of these coatings suffer from weak adhesion or gradual deterioration.^31–34^ Furthermore, only limited investigations have concurrently tailored the dynamic SEI evolution and the solvation structure during battery cycling. Therefore, a synergistic strategy that simultaneously manages the bulk solvation environment and interfacial nucleation behavior is urgently required.

In this work, we introduce a spatiotemporal decoupling concept that distinguishes solvation chemistry from interfacial design. This strategy incorporates two complementary functional agents, PEGDME and FEC, into a simple ZnSO_4_ based electrolyte. A combination of experimental measurements and theoretical computations verifies that PEGDME functions as a robust Lewis base. It forms stable multidentate Zn–O bonds and selectively occupies the primary Zn^2+^ solvation sheath, thereby reducing water activity in the bulk solution. At the same time, PEGDME assumes a flat lying multidentate arrangement on the Zn(002) crystal plane, which reduces the diffusion barrier for Zn adatoms and encourages two dimensional planar growth. By contrast, because of the electron withdrawing nature of its fluorine atoms, FEC exhibits weaker Lewis basicity and therefore does not compete for the inner solvation shell. Instead, it preferentially attaches to step sites and, leveraging its low LUMO energy, reductively decomposes to generate a durable ZnF_2_-rich SEI. This SEI accomplishes two essential tasks. It deactivates defect sites that would otherwise act as favored nucleation spots for dendrites, and it further curbs HER activity. The functions of the two additives are partitioned in space yet synergistic in time. PEGDME operates primarily in the bulk electrolyte and on flat crystal facets, whereas FEC targets step edges. PEGDME remains stable within the solvation sheath, whereas FEC decomposes in a dynamic manner to form the SEI. This spatiotemporal cooperative effect effectively eliminates both the HER and the early stages of dendrite formation. Owing to this multi-dimensional cooperative effect, the resultant Zn anode demonstrates exceptional durability, with symmetric batteries running stably for 1100 hours at 1 mA cm^−2^ and 1 mA h cm^−2^. Asymmetric Zn//Cu batteries deliver a high average CE of 99.2% across 200 cycles. These performance indicators surpass the majority of previously documented systems. Thus, the present study elucidates at the molecular scale the synergistic interplay between coordination driven and decomposition driven additives, while also providing a fresh strategy for disentangling complicated failure mechanisms in aqueous metal battery systems.

## Results and discussion

### DFT calculations reveal the synergistic mechanism of dual additives

**Fig. 1:**
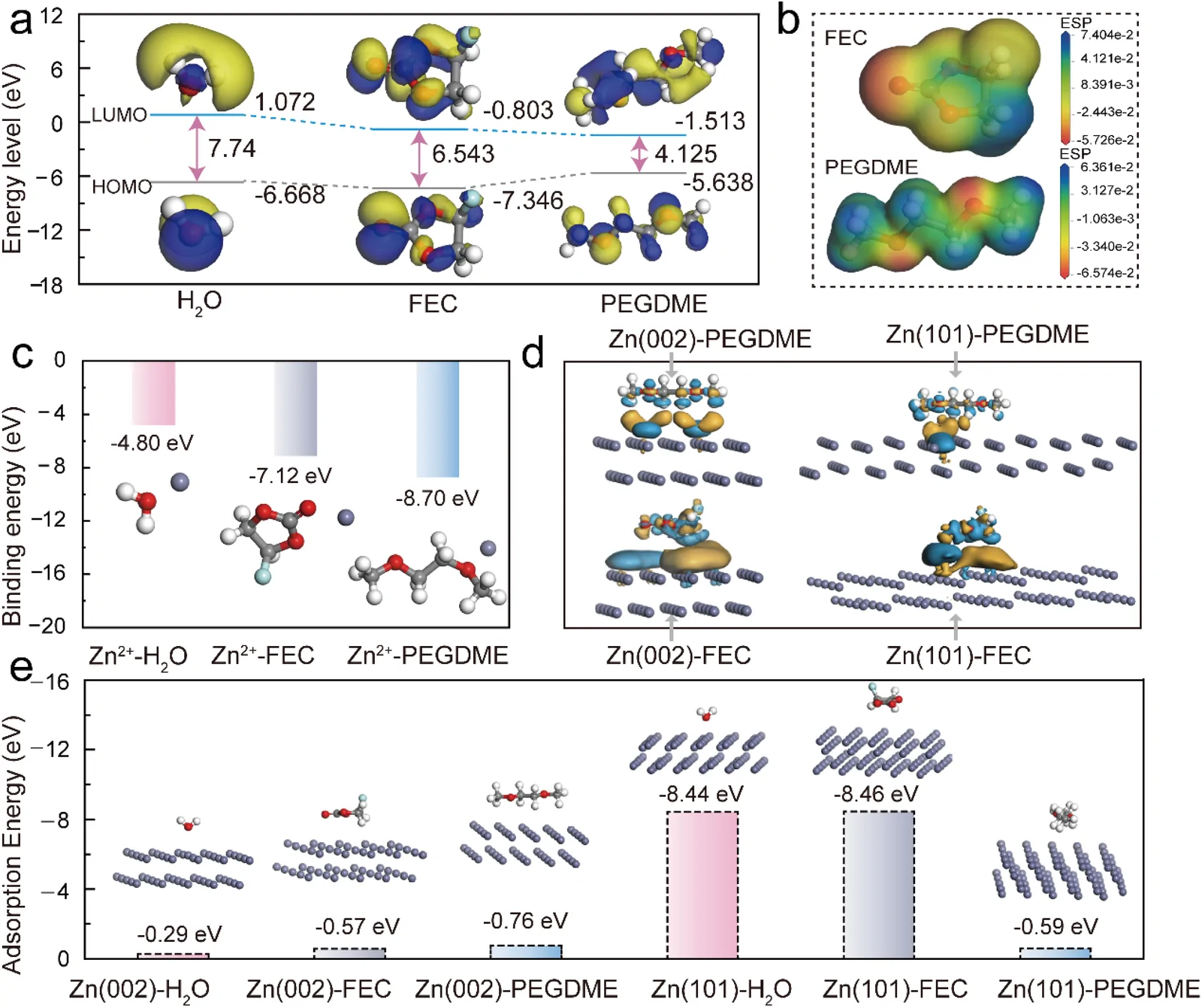
DFT calculations reveal the synergistic mechanism of FEC and PEGDME dual additives. (a) Frontier molecular orbital (HOMO and LUMO) energy levels of H_2_O, FEC, and PEGDME. (b) Electrostatic potential distributions of FEC and PEGDME molecules. (c) Binding energies of Zn^2+^ with H_2_O, FEC, and PEGDME. (d) Differential charge density of PEGDME and FEC adsorbed on Zn(002) and Zn(101) facets. Yellow and blue isosurfaces represent charge accumulation and depletion, respectively. (e) Adsorption energies of H_2_O, FEC, and PEGDME on Zn(002) and Zn(101) facets.

To gain insight into the cooperative action of the FEC/PEGDME additive pair, DFT computations were carried out as an initial step. The HOMO and LUMO energy alignments for H_2_O, FEC, and PEGDME are displayed in Fig. 1a. Relative to H_2_O, both FEC and PEGDME exhibit markedly reduced LUMO levels (−0.803 eV and −1.513 eV, respectively), signifying enhanced electron accepting tendencies and a propensity for preferential reduction at the Zn electrode. PEGDME stands out with the lowest LUMO value, implying the greatest ease of reduction. Additionally, the HOMO levels of FEC and PEGDME lie below that of H_2_O, reflecting superior oxidation tolerance and an expanded electrochemical operating window. Fig. 1b illustrates the electrostatic potential (ESP) mapping for FEC and PEGDME. For FEC, regions of negative electrostatic potential are localized around the carbonyl O and F atoms. In contrast, PEGDME shows a chain like distribution of negative potential along its repeating ether O units. These electron rich domains serve as anchoring sites for robust Zn^2+^ coordination. The strength of these coordination interactions was evaluated *via* binding energy calculations between Zn^2+^ and various ligands, with results compiled in Fig. 1c. PEGDME binds Zn^2+^ with an energy of 8.7 eV, far exceeding the corresponding values for H_2_O (4.8 eV) and FEC (7.12 eV). Thus, PEGDME exhibits the highest Zn^2+^ affinity among the tested species, allowing it to displace coordinated H_2_O and access the inner Zn^2+^ solvation shell. Subsequently, the adsorption characteristics of the additives on Zn crystal facets were examined. Differential charge density maps for PEGDME and FEC adsorbed onto Zn(002) and Zn(101) surfaces are provided in Fig. 1d. For both additives, the extent of charge redistribution is more pronounced on the Zn(002) terraces relative to the Zn(101) step edges. On Zn(002), PEGDME yields contiguous yellow isosurfaces (charge accumulation) across its ether O atoms, accompanied by linked blue zones (charge depletion) on the Zn substrate atoms beneath. This observation is characteristic of multidentate chemical adsorption. In contrast, on Zn(101) the interaction remains limited to individual point contacts. For FEC adsorbed on Zn(002), notable yellow buildup occurs at both the carbonyl O and the F atom, signaling a planar two point attachment geometry accompanied by C–F bond activation during adsorption. On Zn(101), only faint charge redistribution linked to the carbonyl group is detected.

Fig. 1e summarizes the computed adsorption energies for the different species on Zn crystal planes. On the Zn(002) plane, the adsorption energies rank as PEGDME (0.76 eV), FEC (0.57 eV) and H_2_O (0.29 eV). This trend aligns with the differential charge density findings and verifies that the additives preferentially adhere to the (002) terraces. Conversely, on Zn(101), both FEC and H_2_O demonstrate markedly larger adsorption energies (8.46 eV and 8.44 eV) than PEGDME (0.59 eV), revealing particularly strong binding of FEC and water at step sites. PEGDME thus restructures the Zn^2+^ solvation environment *via* robust coordination and selectively adheres to Zn(002) terraces through multidentate chemisorption, facilitating two dimensional Zn migration. FEC plays two distinct roles. Its reduced LUMO level promotes selective reductive breakdown to generate a ZnF_2_ enriched SEI. Meanwhile, its exceptionally strong adhesion to Zn(101) step edges deactivates sites that are susceptible to dendrite nucleation.

### Solvation structure modulation and desolvation kinetics

**Fig. 2:**
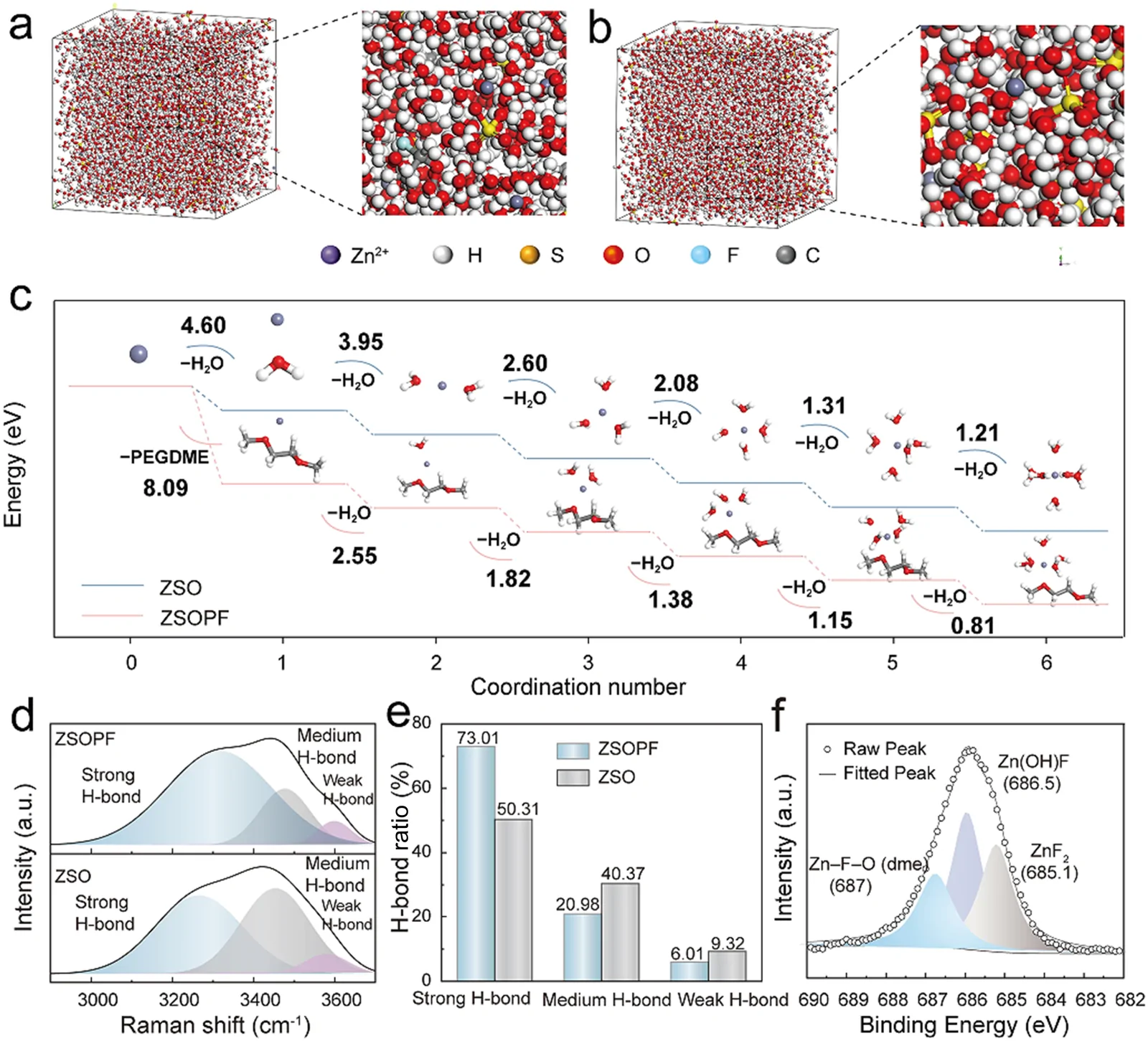
Molecular dynamics (MD) simulations and desolvation energy calculations revealing the solvation structure of the ZSOPF electrolyte. (a) Snapshot of the Zn^2+^ solvation structure in the ZSOPF electrolyte from MD simulations. (b) Snapshot of the Zn^2+^ solvation structure in the pristine ZSO electrolyte from MD simulations. (c) Stepwise desolvation energies of Zn^2+^ in the ZSO and ZSOPF systems. In the ZSOPF system, the removal energies for water molecules are significantly lower than those in the ZSO system. The final removal of the coordinated PEGDME molecule requires a substantially higher energy of 8.09 eV, confirming the ultrahigh stability of the Zn^2+^–PEGDME complex in the bulk electrolyte. (d) Raman spectra of the ZSO and ZSOPF electrolytes with fitted hydrogen bonding curves. (e) Statistical proportions of strong, medium, and weak hydrogen bonds derived from the Raman spectra. (f) High resolution XPS spectra of the cycled Zn anode surface.

To complement the static DFT picture, molecular dynamics (MD) simulations were conducted to directly visualize the time dependent coordination sphere of Zn^2+^.^35^ Representative MD snapshots depicting the Zn^2+^ solvation configuration in the ZSOPF and ZSO electrolytes are displayed in Fig. 2a and b, respectively. The ZSOPF system reveals a markedly altered solvation arrangement. Specifically, PEGDME inserts itself into the primary coordination sphere and binds Zn^2+^*via* its ether O atoms. FEC, in contrast, remains in the outer coordination shell, consistent with its comparatively weaker binding energy and its designated function as an SEI precursor at the interface. Quantitative thermodynamic support for the accelerated Zn^2+^ transport kinetics imparted by the two additives comes from the desolvation energy data presented in Fig. 2c. For the ZSO baseline, desolvation proceeds through six successive H_2_O elimination steps from the Zn(H_2_O)_6_^2+^ complex, demanding energies of 1.21, 1.31, 2.08, 2.60, 3.95, and 4.60 eV. In the ZSOPF system, with PEGDME occupying the inner solvation sheath, the five water removal steps require markedly lower energies, namely 0.81, 1.15, 1.38, 1.82, and 2.55 eV. This consistent per step energy decrease indicates that PEGDME incorporation substantially diminishes the Zn^2+^–H_2_O bond strength, rendering coordinated water more readily detachable. Strikingly, the ultimate elimination of the ligated PEGDME entity demands an extraordinary 8.09 eV, which greatly surpasses the 4.60 eV required for the last water removal in the ZSO case. Such an elevated desolvation barrier substantiates that the bulk medium guarantees sustained coordination throughout ionic migration. When the ion approaches the Zn electrode, the vigorous adsorption of PEGDME onto the Zn(002) terraces fosters lateral Zn movement and directs even deposition, with the coordinated PEGDME eventually liberated or substituted at the interface.

Raman scattering measurements were undertaken to independently confirm the solvation structure alteration.^36^Fig. 2d and e reveal that adding FEC and PEGDME increases the fraction of strong hydrogen bonds from 50.31% to 73.01%, demonstrating that free water reactivity is efficiently curtailed. These spectroscopic observations thus verify the computationally predicted reconfiguration of the solvation sheath. XPS examination of the post cycling Zn anode (Fig. 2f) discloses a clear ZnF_2_ peak at 685.1 eV as well as a Zn–F–O complex feature at 687 eV. These spectral features corroborate both the selective reductive breakdown of FEC into an inorganic ZnF_2_ dominated SEI and the involvement of PEGDME in stabilizing the interfacial region. Taken together, the combined theoretical and experimental evidence delineates a multifaceted cooperative mechanism. PEGDME invades the inner solvation shell, attenuates Zn^2+^–H_2_O bonding, and enhances lateral diffusion across Zn(002) terraces. FEC resides in the outer coordination shell, preferentially reduces to form a ZnF_2_-rich SEI, and passivates the Zn(101) step edges. This spatiotemporal cooperative effect disentangles the deleterious HER and dendrite proliferation processes, thereby profoundly stabilizing the Zn electrode.

### Enhanced interfacial stability and suppressed parasitic reactions

**Fig. 3:**
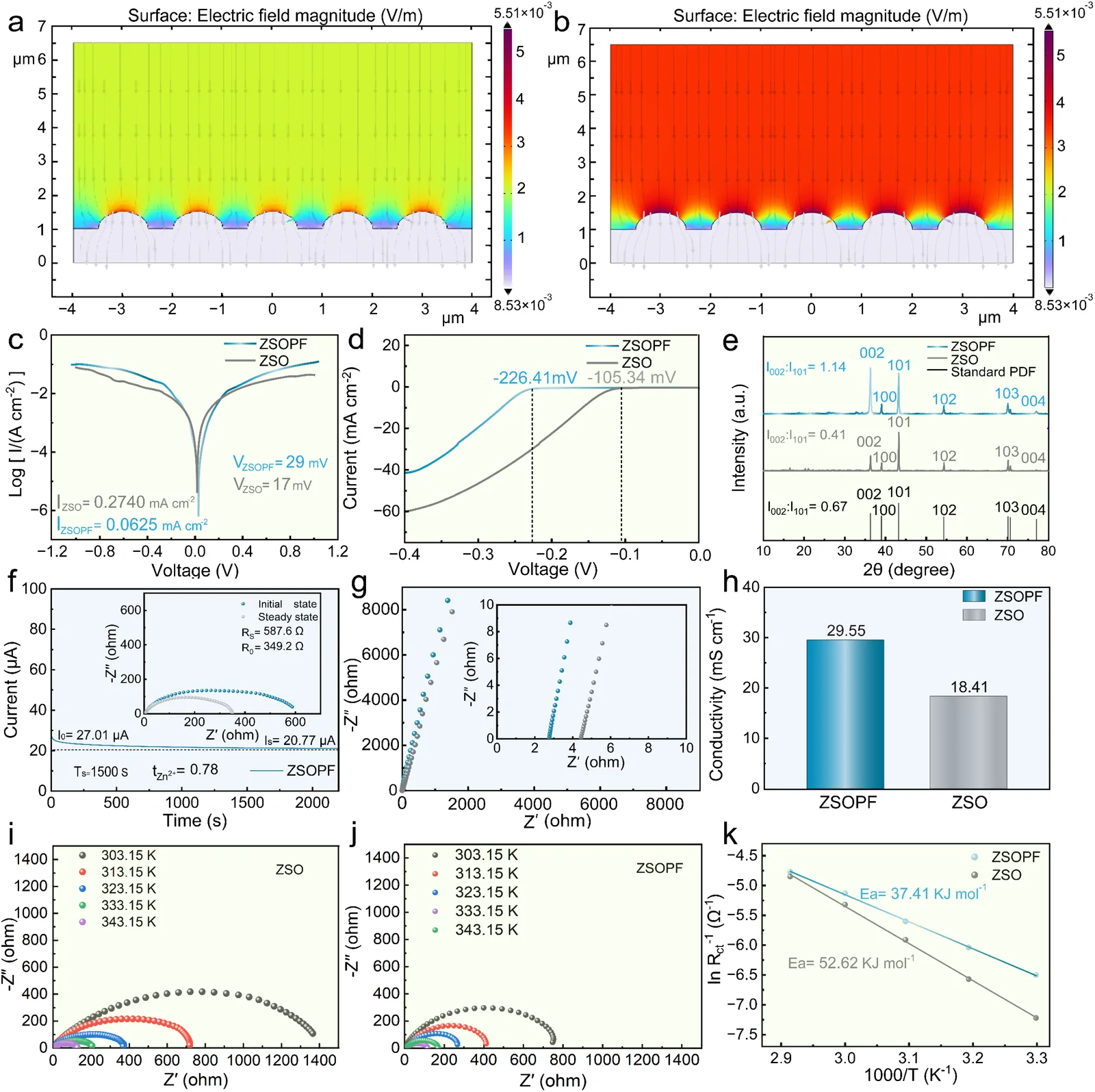
Dual additive enabled enhancement of interfacial stability and suppression of parasitic reactions. (a) and (b) COMSOL simulated electric field mapping on Zn anodes. (a) ZSOPF electrolyte: uniform field distribution across the entire surface and absence of localized hotspots, promoting homogeneous Zn^2+^ flux and dendrite free deposition. (b) ZSO electrolyte: highly nonuniform field with intense hotspots at protrusions and defects, inducing tip effects and accelerating dendrite formation. The color bar indicates normalized electric field intensity. (c) Tafel plots of Zn anodes in ZSO and ZSOPF at 1 mV s^−1^. (d) HER polarization curves of Zn anodes in ZSO and ZSOPF. (e) XRD patterns of Zn anodes after 100 cycles in ZSO and ZSOPF. (f) Chronoamperometry response of a Zn//Zn symmetric battery in ZSOPF at 25 mV overpotential. Inset: impedance spectra before and after polarization. (g) Nyquist plots of ZSO and ZSOPF (inset: ionic conductivities). (h) Ionic conductivity comparison bar chart. (i) and (j) Nyquist plots of Zn//Zn symmetric batteries in ZSO and ZSOPF at 30–70 °C. (k) Arrhenius plots and derived activation energies for Zn^2+^ desolvation. The dual additives markedly lower corrosion current, inhibit the HER, favor Zn(002) textured growth, and reduce the desolvation barrier from 52.62 to 37.41 kJ mol^−1^.

The influence of the dual additives on the spatial distribution of the interfacial electric field was further explored *via* COMSOL Multiphysics modeling.^37^Fig. 3a and b display the calculated electric field profiles at the Zn electrode surface for the ZSOPF and ZSO systems, respectively. Within the unmodified ZSO electrolyte, the electric field is strongly nonuniform, featuring intense hotspots localized around surface asperities and imperfections. Such localized field intensification is widely recognized to expedite Zn^2+^ exhaustion and stimulate dendrite evolution. By marked contrast, the ZSOPF electrolyte displays a highly homogeneous electric field over the whole Zn surface. Incorporating FEC and PEGDME efficiently equalizes the charge distribution at the interface, removing any localized field accumulation. This enhanced field homogeneity, coupled with the previously mentioned lower nucleation overpotential and enlarged electrochemically active surface area, yields the compact and dendrite free Zn morphologies seen in experiments. Tafel polarization experiments were carried out to assess the corrosion resistance of Zn anodes in various electrolytes. Fig. 3c demonstrates that the Zn anode in ZSOPF shows a greatly diminished corrosion current density (0.0625 mA cm^−2^) relative to that in pure ZSO (0.2740 mA cm^−2^). A positive shift in corrosion potential is also observed, signifying that ZSOPF efficiently passivates the Zn surface and improves its thermodynamic stability. LSV measurements were performed to probe HER activity. According to Fig. 3d, the HER onset potential in ZSOPF shifts substantially negatively to 226.41 mV, relative to 105.34 mV in the ZSO case. This outcome clearly indicates that the combined addition of FEC and PEGDME collaboratively inhibits interfacial water reduction, thus expanding the electrochemical stability range. XRD measurements were conducted on Zn anodes harvested after 100 cycles to monitor crystallographic changes.^38^Fig. 3e reveals that the Zn(002)/Zn(101) diffraction peak intensity ratio increases from roughly 0.41 in ZSO to 1.14 in ZSOPF. This substantial enhancement signals a shift to highly textured deposition governed by the close packed (002) plane, a configuration conducive to dense and flat Zn growth. The observed (002) preference arises from two contributions, which are the planar multidentate attachment of PEGDME on (002) terraces that fosters lateral Zn spreading, and the firm anchoring of FEC at (101) step edges that obstructs dendrite nucleation sites.

The Zn^2+^ transference number was measured by chronoamperometry with a constant overpotential of 25 mV. As displayed in Fig. 3f, the ZSOPF electrolyte achieves a high Zn^2+^ transference number of 0.78, substantially higher than that of conventional ZnSO_4_ electrolytes. The current reaches a steady state after approximately 1500 s. In contrast, the chronoamperometry curve of the ZSO electrolyte, shown in Fig. S1, exhibits a continuous current decay without reaching a steady state, indicating severe interfacial instability and continuous parasitic reactions. This high transference number indicates that the composite interface effectively restricts anion migration while facilitating Zn^2+^ transport, ensuring uniform Zn^2+^ flux and suppressing concentration polarization.^39^ The ionic conductivity of different electrolytes was evaluated using stainless steel symmetric batteries. As shown in Fig. 3g and h, the ZSOPF electrolyte exhibits an ionic conductivity of 29.55 mS cm^−1^, significantly higher than that of the pure ZSO electrolyte (18.41 mS cm^−1^). For comparison, the single additive systems show only moderate improvements. As shown in Fig. S2 and S3, the ZSOF electrolyte with FEC alone achieves an ionic conductivity of 21.86 mS cm^−1^, and the ZSOP electrolyte with PEGDME alone reaches 20.02 mS cm^−1^. These values are considerably lower than that of the ZSOPF system, confirming the synergistic effect of the dual additives. This enhancement is attributed to two factors. First, PEGDME reconstructs the solvation sheath, reducing the migration resistance of Zn^2+^. Second, FEC improves the interfacial wettability, facilitating ion transport across the electrode surface. To further probe the Zn^2+^ desolvation kinetics, electrochemical impedance spectroscopy was performed at various temperatures ranging from 30 °C to 70 °C, as shown in Fig. 3i and j. The ZSOPF electrolyte consistently exhibits lower charge transfer resistance than the ZSO electrolyte across the entire temperature range. The activation energy for Zn^2+^ desolvation and diffusion was calculated using the Arrhenius equation. As shown in Fig. 3k, the activation energy decreases from 52.62 kJ mol^−1^ in the ZSO electrolyte to 37.41 kJ mol^−1^ in the ZSOPF electrolyte. This significant reduction demonstrates that the dual additives effectively lower the energy barrier for Zn^2+^ desolvation, accelerating the interfacial reaction kinetics.

### Uniform and dendrite free zinc deposition morphology

**Fig. 4:**
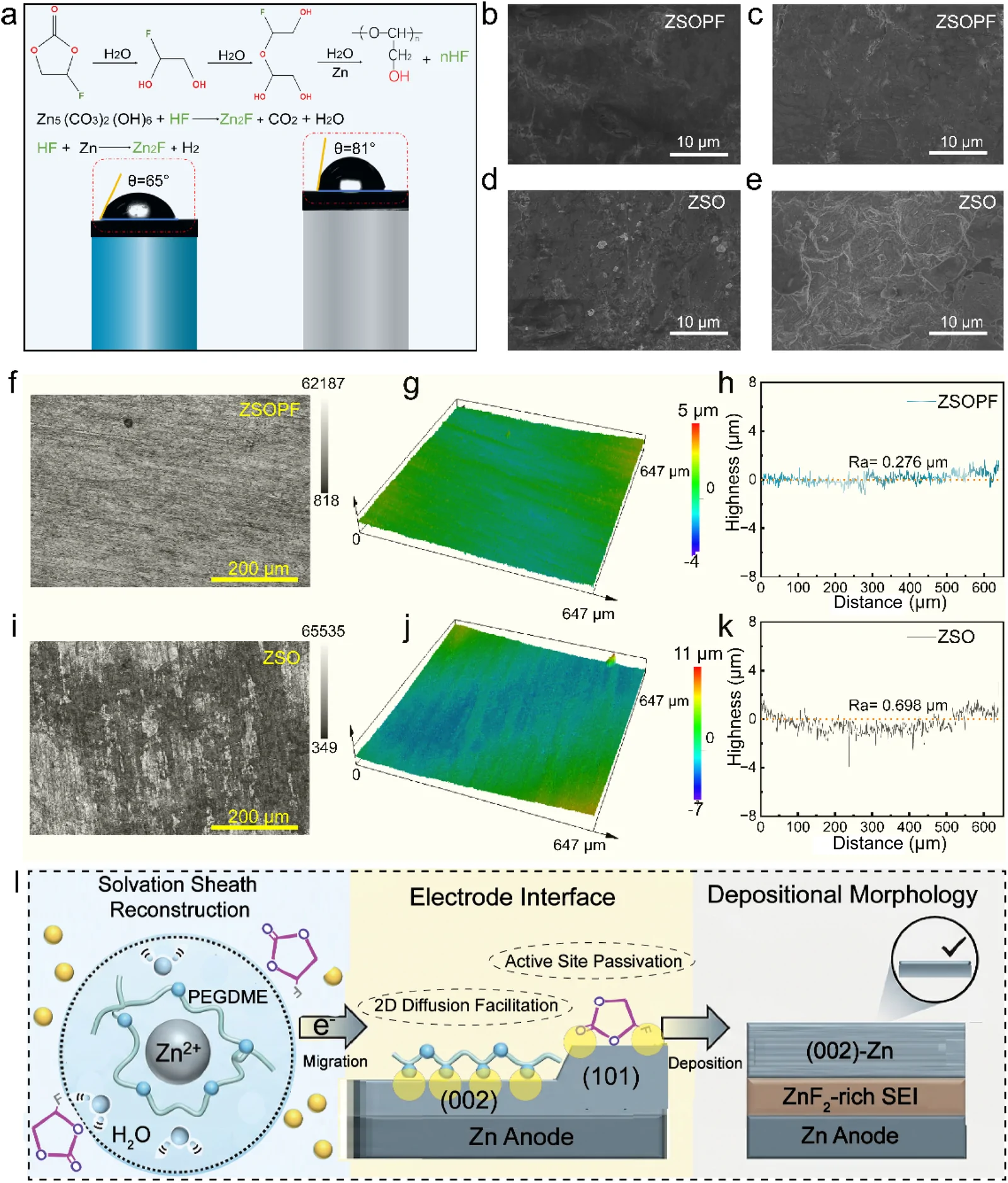
Uniform and dendrite free zinc deposition morphology induced by the dual additives. (a) Contact angle images of ZSO and ZSOPF electrolytes on Zn foil surfaces. (b) and (c) SEM images of Zn deposits after 50 and 100 cycles in ZSOPF electrolyte at 1 mA cm^−2^ and 1 mA h cm^−2^. (d) and (e) SEM images of Zn deposits after 50 and 100 cycles in ZSO electrolyte under the same conditions. (f) and (i) Two-dimensional laser confocal SEM images of Zn anodes after 50 cycles in ZSOPF and ZSO electrolytes. (g) and (j) Corresponding three dimensional LCSM topography images. (h) and (k) Surface height profiles and roughness curves. (l) Schematic illustration of the synergistic mechanism of PEGDME and FEC dual additives for stabilizing the Zn anode. The ZSOPF electrolyte induces dense, smooth, and dendrite free Zn deposition with an average surface roughness of only 0.276 µm, in sharp contrast to the loose and rough morphology with an average roughness of 0.698 µm observed in the ZSO electrolyte.

Contact angle measurements were used to assess electrolyte wettability on Zn anodes.^40^ From Fig. 4a, the ZSOPF system yields a contact angle of 65° on Zn, markedly less than the 81° recorded for ZSO. Dynamic contact angle measurements, shown in Fig. S4, further confirm this enhanced wettability. The ZSOPF electrolyte begins to penetrate the Zn surface within 25 seconds and the contact angle continuously decreases over time, whereas the ZSO electrolyte shows negligible changes even after 50 seconds. This improved wettability ensures uniform ion flux and facilitates the establishment of a stable electrode electrolyte interface. Post cycling Zn deposit morphologies were inspected *via* SEM. Fig. 4b and c demonstrate that after 50 and 100 cycles in ZSOPF, the Zn layers are compact, even, and devoid of dendrites. In sharp contrast, the Zn deposit in the ZSO electrolyte, shown in Fig. 4d and e, is loose, porous, and covered with numerous irregular sharp protrusions. The long-term morphological evolution of the Zn anode was further examined at extended cycle numbers. As shown in Fig. S5 and S6, the Zn anode cycled in the ZSOPF electrolyte maintains a dense and smooth surface even after 150 cycles at both 1 µm and 10 µm scales. In contrast, the Zn anode cycled in the ZSO electrolyte exhibits progressively more severe surface roughening and dendrite accumulation with increasing cycle numbers. These observations demonstrate that the dual additives effectively guide uniform Zn nucleation and growth, suppressing the formation of dendritic structures. LCSM was additionally applied to characterize the surface topography of cycled Zn anodes. Fig. 4f–h display 2D and 3D views of the Zn anode after cycling in ZSOPF. The surface is remarkably flat with only minor undulations, and the average surface roughness is as low as 0.276 µm. In contrast, the Zn anode cycled in the ZSO electrolyte, shown in Fig. 4i–k, exhibits severe height fluctuations and numerous sharp spikes, with an average roughness of 0.698 µm, more than 2.5 times that of the ZSOPF system. These quantitative results unequivocally confirm that the dual additives promote highly uniform Zn deposition and effectively inhibit dendrite growth. Based on the above experimental and theoretical results, we propose a schematic illustration of the synergistic mechanism, as shown in Fig. 4l. In the bulk electrolyte, PEGDME with strong Zn^2+^ affinity enters the primary solvation sheath, partially replacing coordinated water molecules and suppressing water induced side reactions. Upon approaching the Zn electrode surface, a distinct plane selective adsorption behavior occurs. On the Zn(002) terrace, PEGDME adopts a flat lying multi dentate chemisorption configuration, promoting two-dimensional diffusion of Zn adatoms. On the Zn(101) step, FEC preferentially anchors onto active sites, blocking dendrite initiation. This synergistic effect of solvation reconstruction and interfacial adsorption regulation leads to dense, (002) textured, and dendrite free Zn deposition.

### Highly reversible zinc plating and stripping behavior

**Fig. 5:**
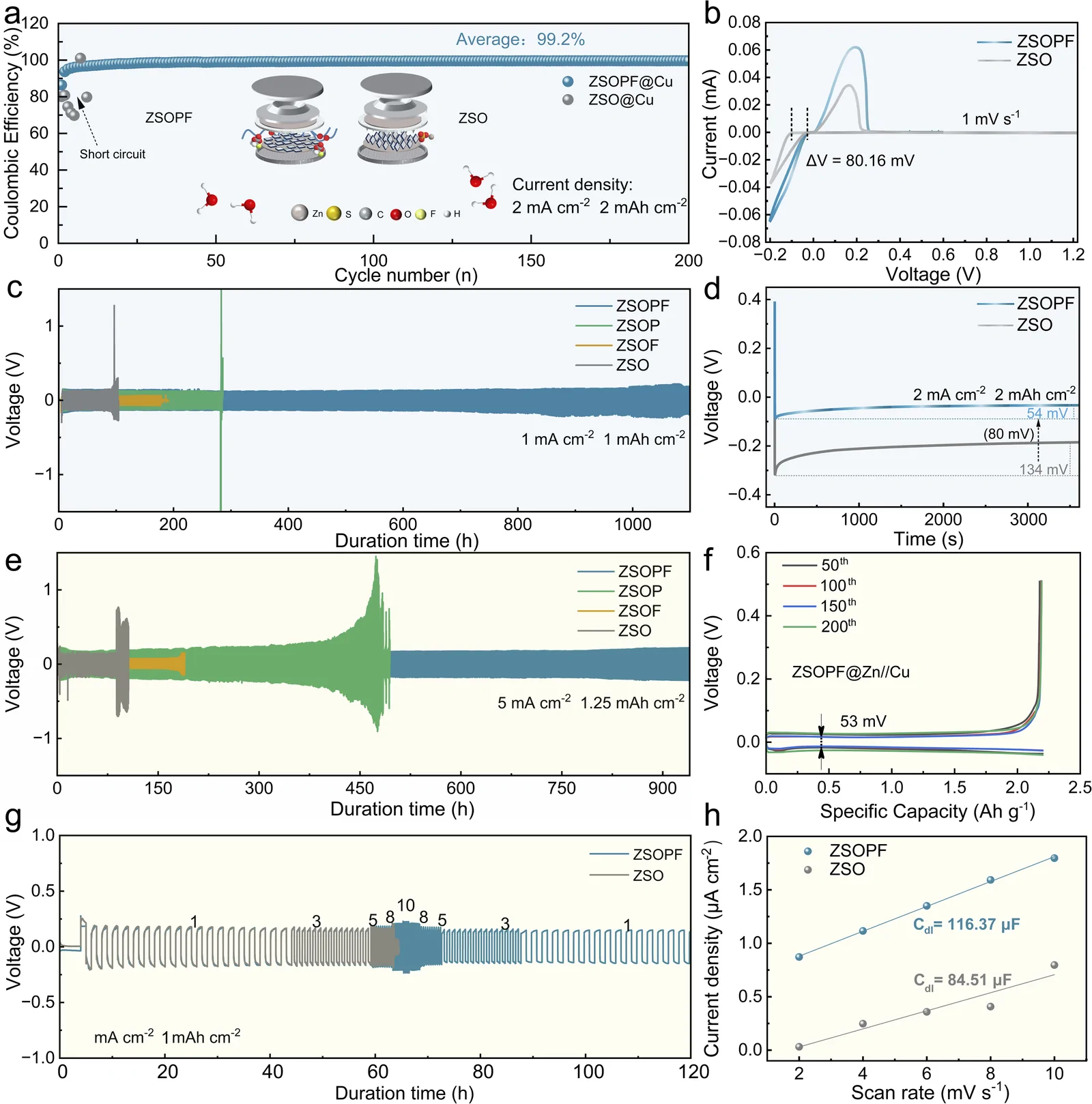
Highly reversible Zn plating and stripping enabled by the dual additives. (a) CE of Zn//Cu asymmetric batteries in ZSO and ZSOPF at 2 mA cm^−2^ and 2 mA h cm^−2^. (b) CV curves of Zn//Cu batteries in ZSO and ZSOPF at 1 mV s^−1^. (c) Long term galvanostatic cycling of Zn//Zn symmetric batteries in ZSO, ZSOP, ZSOF, and ZSOPF at 1 mA cm^−2^ and 1 mA h cm^−2^. (d) Nucleation overpotential profiles of Zn//Cu asymmetric batteries. (e) Long term galvanostatic cycling of Zn//Zn symmetric batteries in ZSO, ZSOP, ZSOF, and ZSOPF at 5 mA cm^−2^ and 1.25 mA h cm^−2^. (f) Galvanostatic charge discharge voltage profiles of Zn//Cu batteries in ZSOPF at selected cycle numbers. (g) Rate capability of Zn//Zn symmetric batteries in ZSO and ZSOPF at current densities of 1–10 mA cm^−2^ with a fixed areal capacity of 1 mA h cm^−2^. (h) Calculated electric double layer capacitances.

Zn plating/stripping reversibility was assessed employing Zn//Cu asymmetric batteries. According to Fig. 5a, the ZSOPF containing battery runs stably for 200 cycles at 2 mA cm^−2^ and 2 mA h cm^−2^, attaining an average CE of 99.2%. The long-term CE of the ZSO electrolyte system is shown in Fig. S7. The pure ZSO electrolyte battery fails after only approximately 10 cycles due to rapid dendrite formation, while the ZSOPF battery maintains stable cycling with consistently high efficiency. In contrast, the battery with the pure ZSO electrolyte exhibits severe fluctuations in CE after only approximately 10 cycles, indicating poor interfacial stability and rapid dendrite formation. CV measurements were carried out to probe Zn nucleation on Cu. Fig. 5b indicates that the nucleation overpotential in ZSOPF is considerably smaller than that in ZSO. This reduction is further confirmed by the galvanostatic charge discharge curves shown in Fig. 5d, where the nucleation overpotential decreases by approximately 80 mV in the ZSOPF system. Specifically, the Zn//Cu asymmetric battery assembled with the ZSOPF electrolyte exhibits a low overpotential of 37 mV at 1 mA cm^−2^ and 1 mA h cm^−2^, as shown in Fig. S8. The 37 mV overpotential achieved in this work is highly competitive when compared with recently reported values (Fig. S9). This low barrier can be attributed to the synergistic effect of PEGDME-mediated solvation reconstruction and FEC-induced interfacial passivation, which collectively lower the energy barrier for Zn^2+^ desolvation and promote uniform nucleation, confirming that the dual additives effectively lower the barrier and promote uniform Zn deposition. CV of Zn//Zn symmetric batteries, shown in Fig. S10 and S11, further confirms the enhanced electrochemical reversibility. The ZSOPF electrolyte exhibits more stable and reproducible CV curves with consistent peak currents over multiple cycles, whereas the ZSO electrolyte shows progressive peak current decay, indicating deteriorating interfacial stability. This consistent trend across two different testing methods demonstrates that the dual additives effectively lower the nucleation barrier, promoting high density and uniform Zn nucleation. The double layer capacitance was measured to evaluate the electrochemically active surface area of the Zn electrode. As shown in Fig. 5h, the ZSOPF electrolyte exhibits a significantly higher double layer capacitance of 116.37 µF compared with 84.51 µF for the ZSO electrolyte. This increase confirms that the dual additives, particularly FEC, greatly improve the surface wettability and expose more active nucleation sites. The combination of reduced nucleation overpotential and increased active sites ensures that the local current density at each nucleation site is substantially lowered, effectively suppressing dendrite formation. The extended cycling durability of Zn//Zn symmetric batteries was tested under multiple conditions. From Fig. 5c, the ZSOPF based battery delivers a lifetime surpassing 1100 hours at 1 mA cm^−2^ and 1 mA h cm^−2^. The pure ZSO electrolyte battery fails after only 93 hours. Notably, the battery with the single FEC additive lasts approximately 180 hours, and the battery with the single PEGDME additive lasts approximately 280 hours, demonstrating that the synergistic effect of the dual additives is far superior to either single component system. As shown in Fig. S12, after 50 cycles at 1 mA cm^−2^, the Zn anode cycled in the ZSO electrolyte exhibited significant blackening and localized pitting corrosion (Fig. S12c), while the ZSOPF-cycled anode remained visually intact with no obvious corrosion (Fig. S12a). After 100 cycles, the Zn anode in the ZSO electrolyte showed extensive and severe corrosion across the entire surface (Fig. S12d), whereas the ZSOPF-cycled anode still maintained a clean and uniform appearance without noticeable degradation (Fig. S12b). These visual observations are fully consistent with the electrochemical results and provide straightforward evidence that the ZSOPF electrolyte effectively suppresses parasitic reactions and corrosion of the Zn anode during long-term cycling. Under more stringent conditions of 5 mA cm^−2^ and 1.25 mA h cm^−2^, shown in Fig. 5e, the ZSOPF electrolyte battery still operates stably for over 950 hours, whereas the pure ZSO and single additive batteries fail rapidly. We systematically screened the additive concentrations by assembling symmetric batteries using ZSOP-0.5F, ZSOP-2F, ZSOF-5P, and ZSOF-10P electrolytes. As shown in Fig. S13, all the single-additive systems exhibited significantly shorter cycle lives compared with the ZSOPF system. At 1 mA cm^−2^, the cycle lives of ZSOP-0.5F, ZSOP-2F, ZSOF-5P, and ZSOF-10P were 220 h, 200 h, 175 h, and 100 h, respectively. At 5 mA cm^−2^, their cycle lives were 110 h, 100 h, 80 h, and 18 h, respectively. Even under more aggressive conditions, the ZSOPF electrolyte maintains superior stability.

As shown in Fig. S14, the symmetric battery with ZSOPF electrolyte operates stably for over 300 hours at 5 mA cm^−2^ and 5 mA h cm^−2^ and 600 hours at 3 mA cm^−2^ and 3 mA h cm^−2^ (Fig. S15). When an ultrathin piece of Zn foil (20 µm) was employed to assemble symmetric batteries, the ZSOPF based batteries delivered a long cycle life of 2000 hours at a current density of 0.1 mA cm^−2^ with an areal capacity of 0.1 mA h cm^−2^ (Fig. S16).

The corresponding voltage profiles, shown in Fig. S17, exhibit stable and low overpotential throughout the entire cycling period. To rule out the possibility that the improved performance simply arises from a concentration effect, we prepared a control electrolyte containing 8 vol% ethylene glycol (EG) at the same total concentration as the ZSOPF system. Symmetric batteries using this ZSOEG electrolyte were assembled and tested under the same conditions as the ZSOPF system. As shown in Fig. S18, the ZSOEG electrolyte delivered cycle lives of only 200 h at 1 mA cm^−2^ and 180 h at 5 mA cm^−2^, significantly inferior to those of the ZSOPF system. These results confirm that the superior performance of the ZSOPF electrolyte originates from the synergistic cooperation between FEC and PEGDME rather than from a mere concentration effect. The rate performance of the Zn//Zn symmetric batteries is shown in Fig. 5g. The ZSOPF electrolyte battery maintains stable voltage profiles across current densities ranging from 1 to 10 mA cm^−2^, while the ZSO electrolyte battery fails at 8 mA cm^−2^. This superior rate capability further confirms that the dual additives not only enhance cycling stability but also significantly improve the reaction kinetics at high current densities.

### Electrochemical performance of Zn//NH_4_V_4_O_10_ batteries

**Fig. 6:**
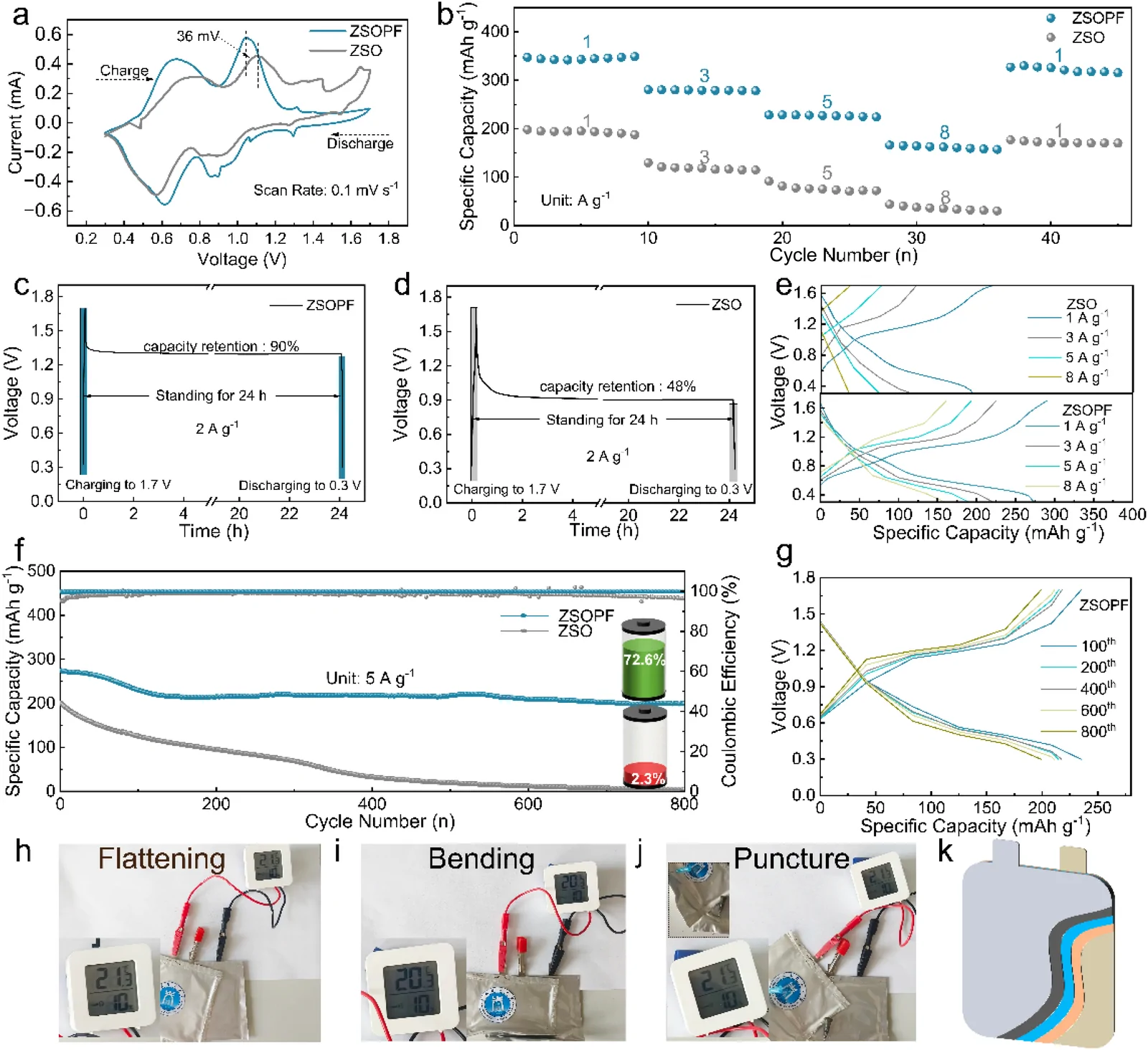
Dual additives enabled excellent full battery performance. (a) CV curves of Zn//NH_4_V_4_O_10_ full batteries in ZSO and ZSOPF at 0.1 mV s^−1^. (b) Rate capability of Zn//NH_4_V_4_O_10_ full batteries at 1–8 A g^−1^. (c) and (d) Storage performance of Zn//NH_4_V_4_O_10_ batteries assembled with ZSOPF and ZSO after 24 h resting at full charge followed by complete discharge. (e) Galvanostatic charge discharge profiles of Zn//NH_4_V_4_O_10_ full batteries at various current densities. (f) Long term cycling stability and capacity retention of Zn//NH_4_V_4_O_10_ full batteries in ZSO and ZSOPF at 5 A g^−1^. (g) Galvanostatic charge discharge curves of the ZSOPF based full battery at selected cycle numbers. (h) Photograph of a temperature sensor powered by a flexible pouch cell assembled with ZSOPF electrolyte. (i) Photograph of the same pouch cell under bending conditions. (j) Photograph of the pouch cell under piercing conditions, with the temperature sensor still operating stably. (k) The schematic diagram of the pouch cell.

To confirm the viability of the ZSOPF formulation, Zn//NH_4_V_4_O_10_ batteries were fabricated. The NH_4_V_4_O_10_ cathode was prepared hydrothermally, with its phase purity verified by XRD. Fig. S19 demonstrates that every diffraction peak matches the standard pattern for NH_4_V_4_O_10_, free of impurity signals, thereby confirming successful material preparation.^41^ CV curves shown in Fig. 6a reveal that the introduction of FEC and PEGDME does not alter the intrinsic redox reactions of the NH_4_V_4_O_10_ cathode, as the peak positions remain largely unchanged. However, the ZSOPF electrolyte battery exhibits a smaller polarization potential difference, indicating lower reaction resistance and faster charge transfer kinetics. Electrochemical impedance spectroscopy (EIS) of the full batteries is shown in Fig. S20. The ZSOPF electrolyte battery exhibits a substantially lower charge transfer resistance than the ZSO electrolyte battery, confirming that the dual additives facilitate faster interfacial reaction kinetics. The rate capability of the full batteries was evaluated at current densities ranging from 1 to 8 A g^−1^. As shown in Fig. 6b, the ZSOPF electrolyte battery delivers consistently higher specific capacities at all current densities compared with the ZSO electrolyte battery. Remarkably, when the current density returns to 1 A g^−1^ after high-rate cycling, the ZSOPF battery recovers approximately 92% of its initial capacity, demonstrating excellent structural stability and reaction reversibility.^42^ As shown in Fig. 6e, the battery in the ZSOPF electrolyte always maintains a relatively high discharge specific capacity, while the capacity of the ZSO system significantly decreases as the rate increases. Meanwhile, the voltage gap between the charge and discharge curves of the ZSOPF system is smaller and the platform is more stable, indicating that its interface impedance is lower and Zn^2+^ transmission is smoother, thereby demonstrating superior rate performance and electrochemical reversibility. The self-discharge behavior was assessed by monitoring the capacity retention after a 24 hour resting period at a fully charged state.^43^ As shown in Fig. 6c and d, the ZSOPF electrolyte battery retains 90% of its initial capacity, significantly higher than the 48% retention observed for the ZSO electrolyte battery. This result confirms that the dual additives effectively suppress zinc corrosion and hydrogen evolution even during idle periods.

The long-term cycling stability of the full batteries was evaluated at a high current density of 5 A g^−1^. As shown in Fig. 6f, the ZSOPF electrolyte battery delivers an initial specific capacity of 274.5 mA h g^−1^ and maintains a capacity retention of 78.6% after 800 cycles, corresponding to a remaining capacity of 200.1 mA h g^−1^. In stark contrast, the ZSO electrolyte battery retains only 2.3% of its initial capacity after the same number of cycles. The galvanostatic charge discharge curves at different cycle numbers, shown in Fig. 6g, demonstrate that the ZSOPF battery maintains stable voltage platforms with minimal polarization even after 800 cycles. In contrast, the ZSO electrolyte battery, shown in Fig. S21, exhibits rapidly increasing polarization and capacity decay with increasing cycle number. The charge discharge plateaus become progressively separated, indicating severe reaction kinetics deterioration and poor interfacial stability. Finally, to demonstrate the practical potential of our strategy, we assembled a flexible pouch cell using the ZSOPF electrolyte to power a temperature sensor; the schematic diagram of the pouch cell is shown in Fig. 6k. The pouch cell maintains stable operation even under bending and piercing conditions. As additional demonstrations of practical utility, the ZSOPF based pouch cell can stably power a CUST patterned LED panel, as shown in Fig. S22, and maintains a stable open circuit voltage of 1.23 V, as shown in Fig. S23. The assembled pouch cell delivers a capacity retention of 90.35% after 130 cycles at a current density of 2 A g^−1^, confirming its stable rechargeable operation (Fig. S24). These visual validations further underscore the practical application potential of our dual additive strategy. The sensor continues to function normally without any observable brightness decay, providing direct visual evidence for the excellent mechanical flexibility and safety of the ZSOPF system under realistic working conditions (Fig. 6h–j). This demonstration highlights the promising application prospects of our dual additive strategy in flexible and wearable energy storage devices.^44^

## Conclusions

In summary, we have developed a spatiotemporal decoupling approach capable of simultaneously addressing the coupled problems of hydrogen evolution and Zn dendrite formation in aqueous zinc ion batteries. By introducing PEGDME and FEC as complementary components into a simple ZnSO_4_ electrolyte, we achieve synergistic regulation across both spatial and temporal dimensions. DFT calculations reveal that PEGDME possesses the highest binding energy with Zn^2+^, enabling it to enter the primary solvation sheath and weaken Zn^2+^–H_2_O coordination. Desolvation energy calculations further confirm that PEGDME forms an ultra-stable complex in the bulk electrolyte while lowering the energy barriers for water removal. Leveraging its strong Lewis basicity and multidentate coordination capability, PEGDME preferentially enters the primary solvation sheath of Zn^2+^ to suppress water activity in the bulk electrolyte. Simultaneously, it adopts a flat lying adsorption configuration on the Zn(002) facet, promoting two dimensional surface diffusion of adatoms. Meanwhile, owing to its low LUMO level and weak Lewis basicity, FEC selectively anchors at electrode defect sites and undergoes preferential reductive decomposition, forming a dynamic ZnF_2_ rich SEI. This SEI passivates active sites, further suppresses the hydrogen evolution reaction, and guides uniform zinc deposition. This functional decoupling mechanism, wherein PEGDME governs solvation regulation and surface diffusion while FEC handles defect passivation and interface stabilization, fundamentally breaks the coupling between hydrogen evolution and dendrite nucleation. Consequently, the Zn//Zn symmetric battery achieves stable cycling for over 1100 hours at 1 mA cm^−2^ and 1 mA h cm^−2^ and maintains a low overpotential of 37 mV. The Zn//Cu asymmetric battery delivers an average CE of 99.2% over 200 cycles. Moreover, the Zn//NH_4_V_4_O_10_ full battery retains 78.6% of its initial capacity after 800 cycles at a high current density of 5 A g^−1^. Thus, this investigation not only clarifies at the molecular level the cooperative interplay between coordination oriented and decomposition oriented additives but also furnishes a generalizable spatiotemporal engineering blueprint for stabilizing metal anodes in aqueous rechargeable battery systems.

## Author contributions

Yu-Xuan Xiao and Si-Ze Wang contributed equally to this work. Yu-Xuan Xiao performed the material synthesis, electrochemical measurements, and data analysis, and drafted the initial manuscript. Si-Ze Wang conducted the density functional theory calculations, molecular dynamics simulations, and desolvation energy calculations, and contributed to the mechanistic interpretation. Han-Hao Liu assisted with the theoretical calculations and data analysis. Zhen-Yi Gu and Xiao-Tong Wang participated in the electrochemical characterization and data curation. Jia-Lin Yang contributed to the Raman and XPS measurements. Long-Xin Zhang and Wen-Ze Luo provided valuable discussions on the solvation structure and interfacial mechanism. Hongbo Zhang, Xinlu Wang and Jinxian Wang contributed to the materials characterization and manuscript revision. Yang Su, Bin Fu and Xing-Long Wu supervised the project, conceptualized the research idea, analyzed the data, and revised the manuscript. All authors reviewed and approved the final version of the manuscript.

## Conflicts of interest

There are no conflicts to declare.

## Supplementary Material

SC-017-D6SC04691F-s001

## Data Availability

The data supporting the findings of this study are available within the article and its supplementary information (SI). Supplementary information: detailed experimental procedures (electrolyte preparation, material synthesis, electrode fabrication, and characterization methods), theoretical calculation details (DFT and MD simulation parameters), additional electrochemical data (impedance spectra, ionic conductivity, Coulombic efficiency, overpotential comparison, and long-term cycling performance under various conditions), figures (Fig. S1–S24), and optical images of cycled Zn anodes and pouch cell demonstrations. See DOI: https://doi.org/10.1039/d6sc04691f.
