# Supplementary material for: Spatiotemporal synergy of a coordination decomposition bifunctional strategy for ultra-stable aqueous zinc anodes
Source: Chem Sci. 2026 Jul 3. Online ahead of print. doi: 10.1039/d6sc04691f (PMC13359085; doi:10.1039/d6sc04691f)
Supplement: SC-OLF-D6SC04691F-s001 [file SC-OLF-D6SC04691F-s001.pdf]

Supplementary Information (SI) for Chemical Science.
This journal is © The Royal Society of Chemistry 2026

Supporting Information

Spatiotemporal Synergy of Coordination Decomposition Bifunctional Strategy for Ultra Stable Aqueous Zinc Anodes

Yu-Xuan Xiao†, Si-Ze Wang†, Yang Su*, Han-Hao Liu, Zhen-Yi Gu, Xiao-Tong Wang, Jia-Lin Yang, Bin Fu*, Long-Xin Zhang, Wen-Ze Luo, Jujun Yuan, Hongbo Zhang, Xinlu Wang, Jinxian Wang and Xing-Long Wu*

1. Experimental Section

1.1 Materials

All chemicals were acquired from commercial sources and used without further purification. $ZnSO_4\cdot7H_2O$ (99%), FEC (99%), and PEGDME (average Mn≈500) were supplied by Aladdin Reagent and Macklin Biochemical. NH_4VO_3 (99%), $H_2C_2O_4\cdot2H_2O$ (99.5%), anhydrous NMP (99.5%), and PVDF were purchased from Sigma Aldrich or local vendors. Zinc foil (thickness: 0.08 mm, 99.9%) and copper foil (thickness: 0.02 mm, 99.9%) were purchased from commercial vendors and used as received after cleaning with dilute acid and deionized water to remove the surface oxide layer. Carbon paper (hydrophilic treated, TGPH060H) was used as the cathode current collector. All aqueous solutions were prepared using deionized water. All chemicals were used without further purification unless otherwise specified.

1.2 Preparation of Electrolytes

A 2 M $ZnSO_4\cdot7H_2O$ solution prepared in deionized water served as the baseline electrolyte, referred to as ZSO. The modified electrolyte (denoted ZSOPF) was formulated by adding specified additive volume percentages relative to the final aqueous mixture. Specifically, 100 μL of FEC (1 vol%) and 700 μL of PEGDME (7 vol%) were introduced into deionized water. The mixture was then magnetically stirred overnight at room temperature to ensure complete mixing and pre coordination. Subsequently, 2.8756 g of $ZnSO_4\cdot7H_2O$ was added to the pre-mixed solution to reach the final 2 M concentration. For comparative investigation, control electrolytes containing 1 vol% FEC were prepared via identical procedures (ZSOF, 2 M $ZnSO_4$ and 1

vol% FEC; ZSOF-5P, 2 M $ZnSO_4$ and 1 vol% FEC and 5 vol% PEGDME; ZSOF-10P, 2 M $ZnSO_4$ and 1 vol% FEC and 10 vol% PEGDME). A series of control electrolytes with various PEGDME contents including 7 vol% PEGDME were also synthesized (ZSOP, 2 M $ZnSO_4$ and 7 vol% PEGDME; ZSOP-0.5F, 2 M $ZnSO_4$ and 7 vol% PEGDME and 0.5 vol% FEC; ZSOP-2F, 2 M $ZnSO_4$ and 7 vol% PEGDME and 2 vol% FEC). The electrolyte containing 8 vol% EG was also synthesized (ZSOEG, 2 M $ZnSO_4$ and 8 vol% EG).

1.3 Synthesis of $NH_4V_4O_{10}$ Cathode Material

A hydrothermal method was adopted for synthesizing the $NH_4V_4O_{10}$ cathode. In a typical run, 0.9524 g of NH_4VO_3 and 1.008 g of $H_2C_2O_4 \cdot 2H_2O$ were dissolved in 80 mL of deionized water with continuous magnetic agitation at 60°C for 30 min, forming a transparent solution. This solution was then placed into a 100 mL Teflon lined stainless steel autoclave and heated at 180°C for 24 h. After the hydrothermal reaction, the autoclave was allowed to cool naturally to room temperature. The obtained precipitate was collected by centrifugation, washed alternately with deionized water and absolute ethanol several times, and finally dried under vacuum at 60°C for 12 hours to yield the final $NH_4V_4O_{10}$ product.

1.4 Electrode Fabrication

Cathode preparation: A conventional slurry coating process was used to fabricate working electrodes for full batteries. The active powder ($NH_4V_4O_{10}$), conductive Super P carbon black, and PVDF binder were mixed at a weight ratio of 7:2:1, using NMP as the dispersing agent. The resulting homogeneous slurry was cast uniformly onto hydrophilic carbon paper current collectors with a doctor blade. The coated electrodes

were initially dried in a forced-air oven at 60°C to remove surface solvent, followed by further drying under vacuum at 80°C for 12 hours. The mass loading of the active material on the electrodes was precisely controlled within the range of 1.0-1.5 mg cm^{-2}. The dried electrodes were subsequently cut into circular discs with a diameter of 12 mm for battery assembly.

Anode Preparation: Commercial Zn foil (0.08 mm thickness) was first polished with fine sandpaper to remove the native oxide layer and surface impurities. It was then rinsed with ethanol and deionized water before being punched into 12 mm diameter discs for use in symmetric, asymmetry, and full batteries.

1.5 Material Characterization

Microscopic and spectroscopic characterization: A Hitachi SU8010 field emission scanning electron microscope (FESEM) was employed to examine the surface morphology and microstructural changes of cycled Zn anodes. A Keyence VK X250 laser confocal scanning microscope (LCSM) was used to obtain three dimensional surface topographs and roughness values (Ra). Crystallographic information of the cycled Zn electrodes and pristine $NH_4V_4O_{10}$ powder was obtained by X-ray diffraction (XRD, Rigaku SmartLab, Japan) with Cu Kα radiation (λ=1.5406 Å) in the 2θ range of 5° to 80°. Raman spectra were collected on a confocal Raman spectrometer (Horiba LabRAM HR Evolution, France) with a 532 nm laser excitation source to investigate the hydrogen bonding environment and solvent-ion interactions in the electrolytes. X-ray photoelectron spectroscopy (XPS) measurements were performed on a Thermo Scientific K-Alpha spectrometer (USA) equipped with a monochromatic Al Kα source

to analyze the chemical composition of the solid electrolyte interphase (SEI) layer formed on the cycled Zn surface. All XPS spectra were calibrated based on the adventitious C 1s peak at 284.8 eV.

Wettability and Physical Properties: The contact angle (CA) of different electrolytes on Zn foil was measured using a Krüss DSA100 drop shape analyzer (Germany) at ambient temperature. A droplet of 5 μL was dispensed onto the substrate, and the image was captured immediately after stabilization (typically within 1-2 s). Dynamic CA evolution was monitored over a period of 50 s to evaluate electrolyte spreading kinetics. Ionic conductivity of the electrolytes was determined via electrochemical impedance spectroscopy (EIS) using a stainless steel (SS) blocking electrode battery (SS//SS). The measurement frequency ranged from 100 kHz to 1 Hz with an amplitude of 5 mV.

1.6 Electrochemical Measurements and Battery Assembly

All electrochemical tests were conducted using standard CR2032 coin-type batteries assembled in ambient air. Whatman glass microfiber filters (GF/D) were used as separators, and the electrolyte volume was fixed at 120 μL per battery to ensure consistent wetting conditions.

Battery Testing Systems: Galvanostatic charge-discharge (GCD) cycling tests for Zn//Zn symmetric batteries, Zn//Cu asymmetric half-batteries, and Zn//$NH_4V_4O_{10}$ full batteries were performed on a Neware battery testing system (CT-4008, Shenzhen, China) within the voltage ranges specific to each test configuration.

Electrochemical Workstation Analysis: Cyclic voltammetry (CV), Tafel plots

(corrosion measurement), linear sweep voltammetry (LSV) for hydrogen evolution reaction (HER) assessment, chronoamperometry (CA) for Zn^{2+} transference number evaluation, and Electrochemical Impedance Spectroscopy (EIS) were all performed on a CHI760E electrochemical workstation (Chenhua Instruments, Shanghai, China).

2. Theoretical Calculations

Spin polarized DFT computations were carried out with the DMol3 code. The exchange correlation functional was treated using the GGA in its PBE parametrization. Core electrons were described via the DSPP approach, while valence orbitals were expanded using DNP basis sets. A Monkhorst Pack grid of 1×1×1 k points was employed for Brillouin zone sampling, and dispersion forces were handled with the Grimme DFT D correction. Self-consistent field convergence was set to 1.0×10^{-6} Ha. Geometry optimizations continued until energy changes fell below 5.0×10^{-5} Ha, forces below 0.004 Ha $Å^{-1}$, and displacements below 0.005 Å. Calculation of zinc ion desolvation energy: The calculations of the stepwise desolvation energy for different solvation structures were carried out by DMol3 program in Materials Studio. Geometric optimization and desolvation energy calculations were performed by GGA/PBE functional. The desolvation energy (ΔE_{ZnAxBy}) is defined as:

$$\Delta E_{ZnAxBy} = E_{ZnA(x-1)By} + E_{A} - E_{ZnAxBy}$$

where E_{ZnAxBy} represents the energy of the zinc ion complex with x solvent molecules (or anions) A and y solvent molecules (or anions) B, $E_{ZnA(x-1)By}$ is the energy of the remaining solvation structure after removing one molecule or ion A, and E_{A} is the energy of an isolated solvent molecule (or anion) A.

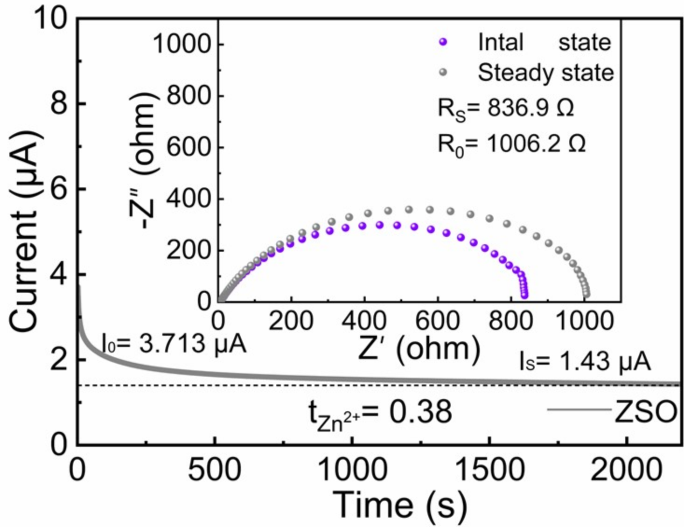

Fig. S1. The chronoamperometry curve in ZSO electrolyte.

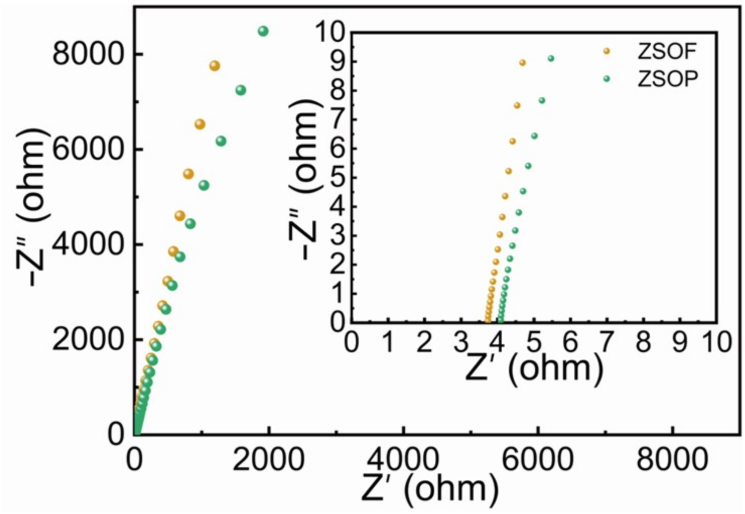

Fig. S2. Impedance spectra of ZSOP and ZSOF electrolytes.

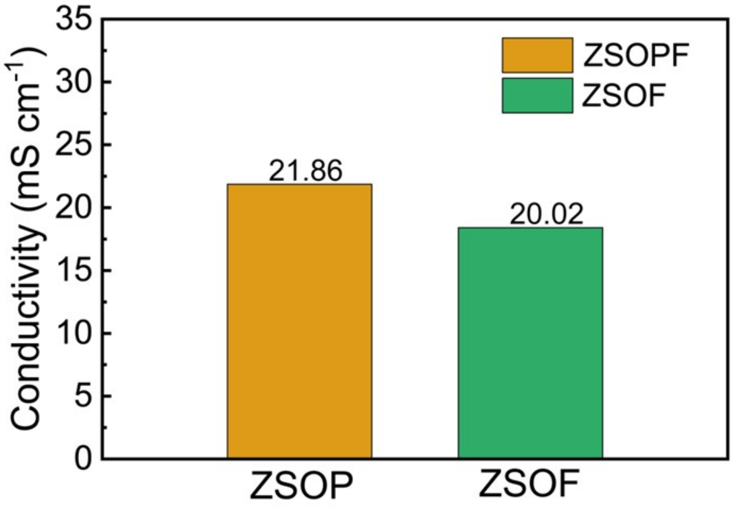

Fig. S3. The bar chart of ionic conductivity of ZSOP and ZSOF electrolytes.

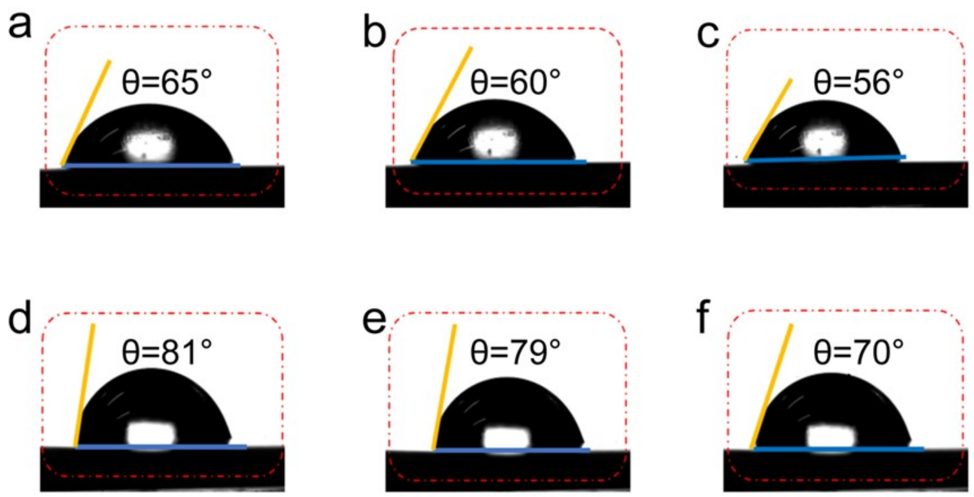

Fig. S4. Schematic diagrams of contact Angle changes at ZSOPF (a,b,c) and ZSO (d,e,f) test times of 0s, 25s, and 50s respectively.

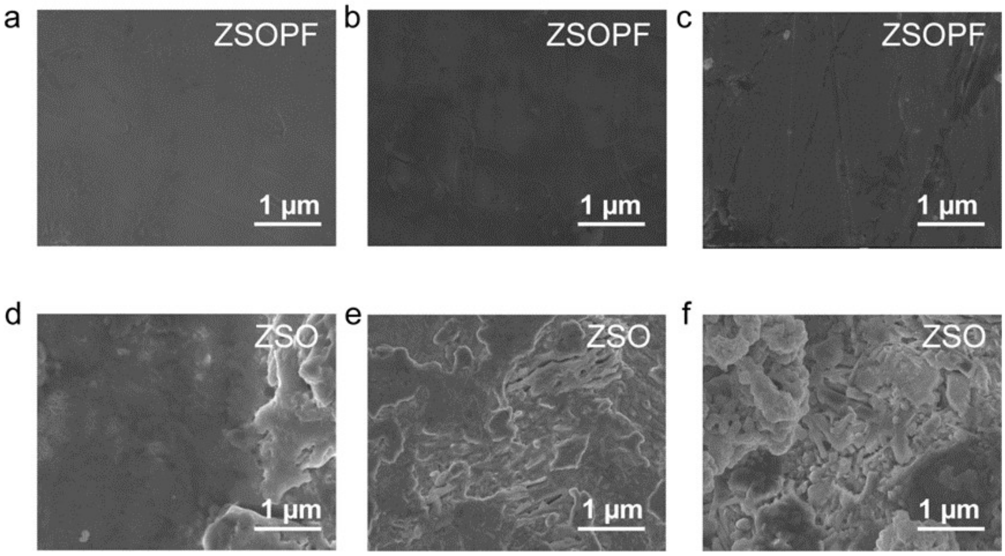

Fig. S5. SEM morphological images of Zn//Zn after cycling 50, 100, and 150 times in ZSOPF (a,b,c) and ZSO (d,e,f)

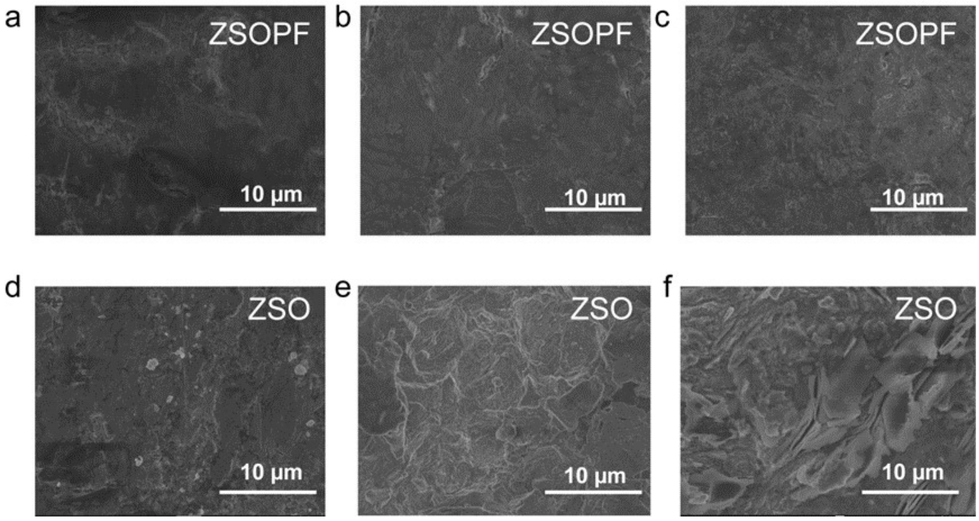

Fig. S6. SEM morphological images of Zn//Zn after cycling 50, 100, and 150 times in ZSOPF (a,b,c) and ZSO (d,e,f).

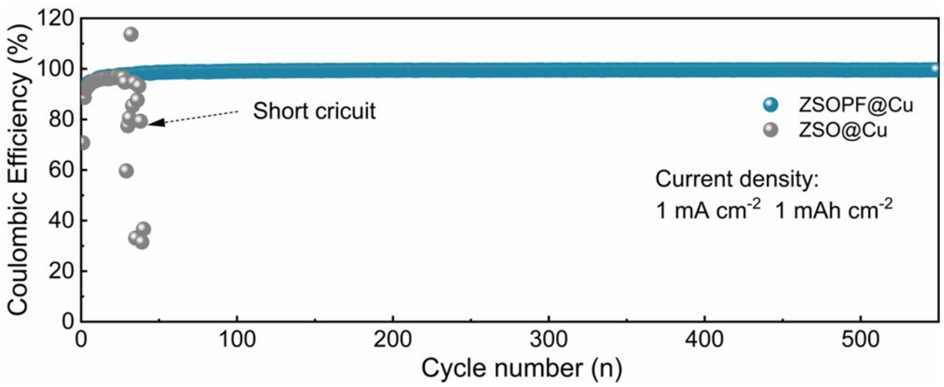

Fig. S7. The Coulombic efficiency of the Zn//Cu asymmetric zinc battery assembled with ZSO and ZSOPF electrolytes under the conditions of 1 mA cm^{-2} and 1 mAh cm^{-2}.

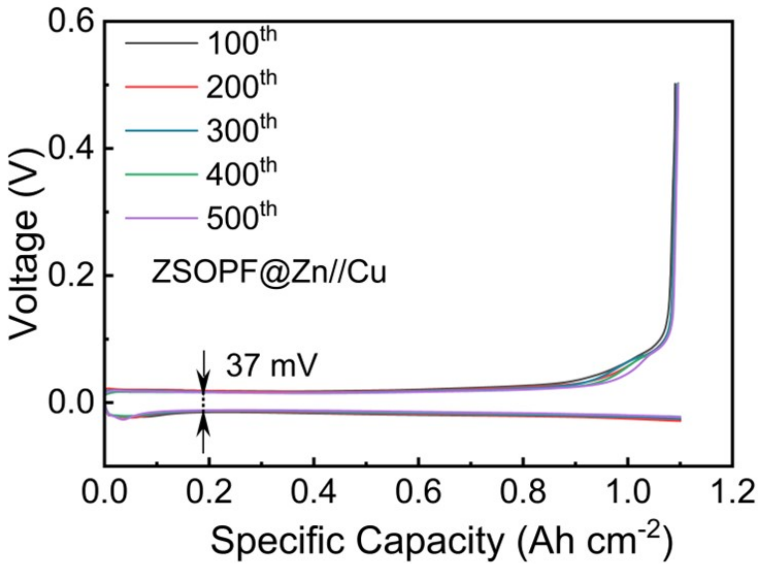

Fig. S8. The overpotential of the Zn//Cu asymmetric battery assembled with ZSOPF electrolyte under the conditions of 1 mA cm^{-2} and 1 mAh cm^{-2}.

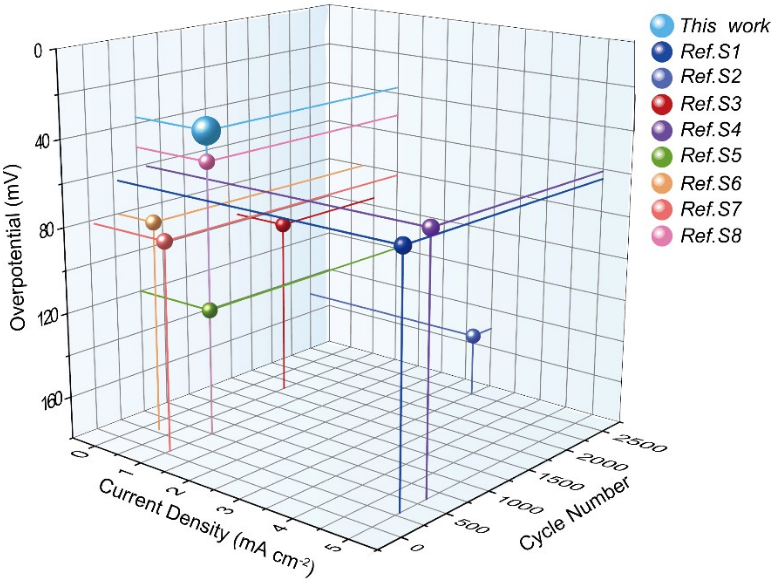

Fig. S9. Comparison of overpotentials between this work and previously reported studies.[1-8]

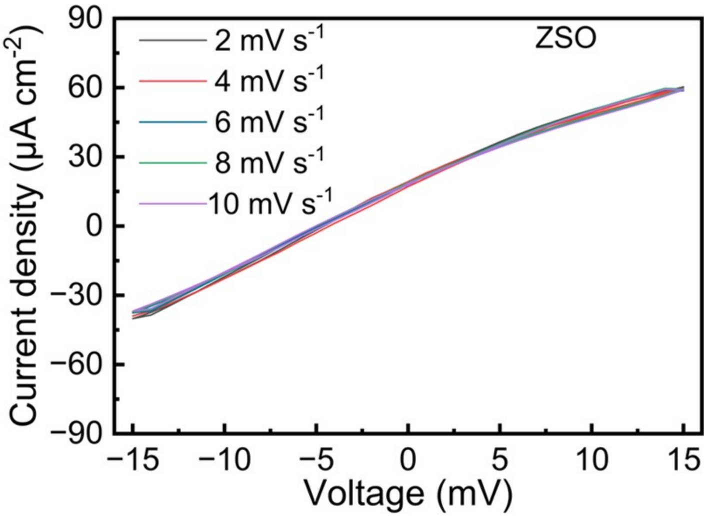

Fig. S10. CV curve of the Zn//Zn symmetric battery in the ZSO electrolyte.

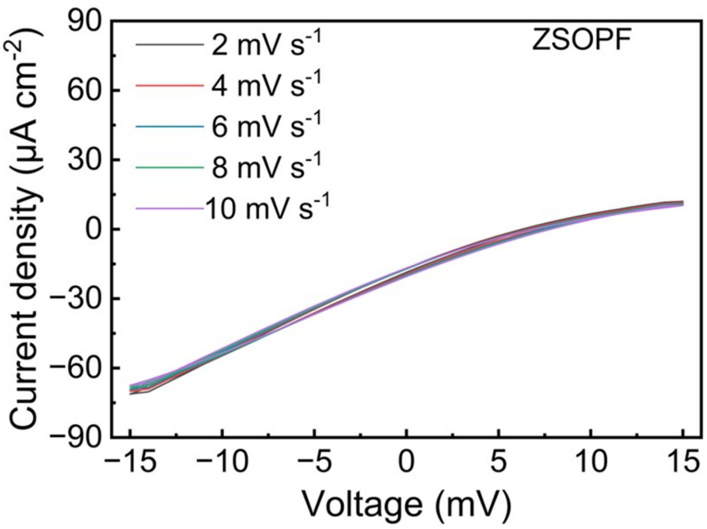

Fig. S11. CV curve of the Zn//Zn symmetric battery in the ZSOPF electrolyte.

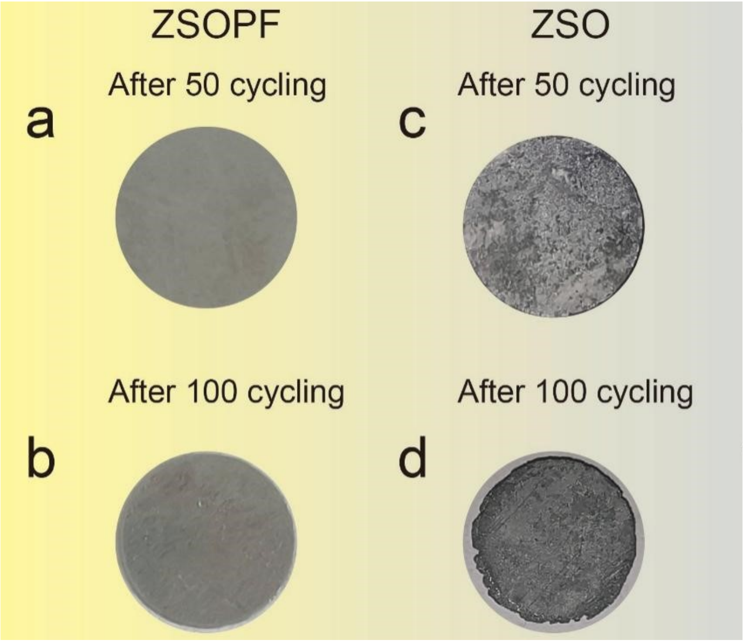

Fig. S12. Optical images of ZSOPF (a) and ZSO (c) after 50 plating/stripping cycles at 1 mA cm^{-2}. Optical images of ZSOPF (b) and ZSO (d) after 100 plating/stripping cycles at 1 mA cm^{-2}.

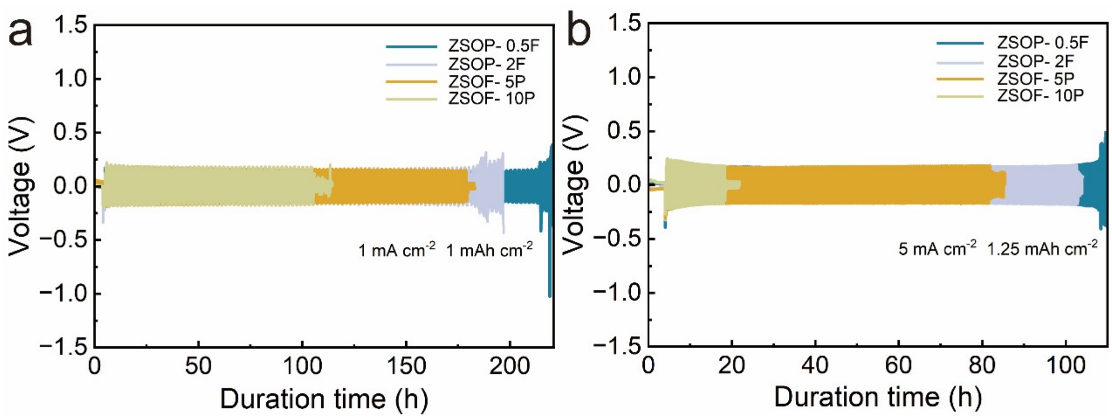

Fig. S13. Galvanostatic cycling of Zn//Zn symmetric batteries in ZSOP-0.5F, ZSOP-2F, ZSOF-5P, and ZSOF-10P at (a) 1 mA cm^{-2} and 1 mAh cm^{-2} and (b) 5 mA cm^{-2} and 1.25 mAh cm^{-2}.

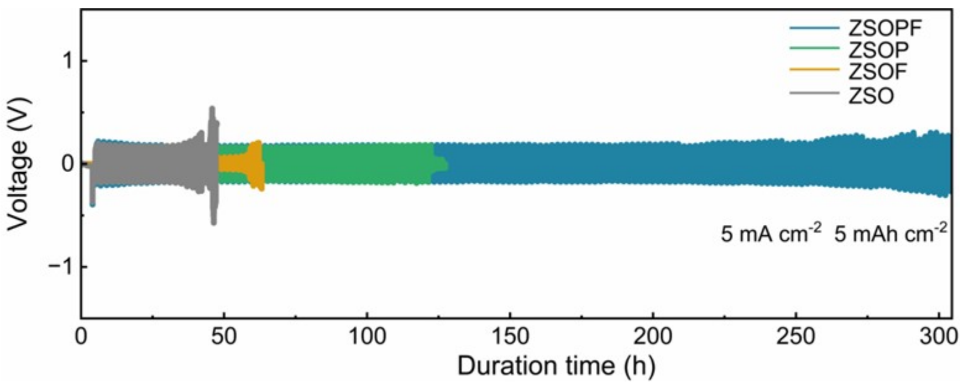

Fig. S14. Under the conditions of 5 mA cm^{-2} and 5 mAh cm^{-2}, the long-term constant current cycling of the Zn//Zn symmetric battery assembled with ZSO, ZSOP, ZSOF and ZSOPF electrolytes.

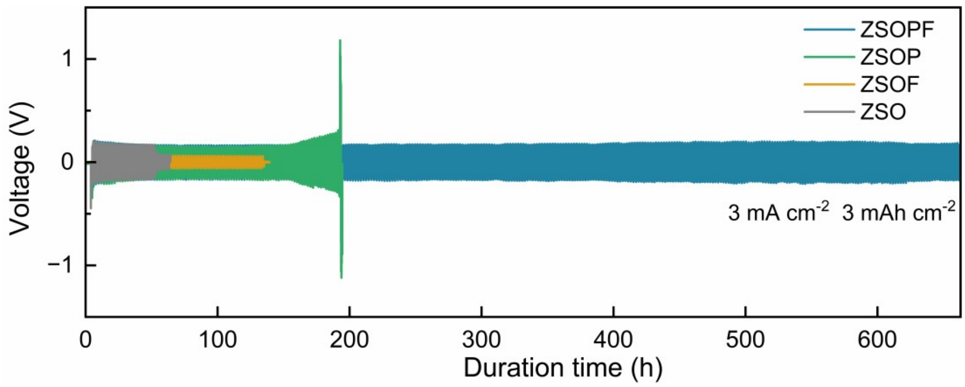

Fig. S15. Under the conditions of 3 mA cm^{-2} and 3 mAh cm^{-2}, the long-term constant current cycling of the Zn//Zn symmetric battery assembled with ZSO, ZSOP, ZSOF and ZSOPF electrolytes.

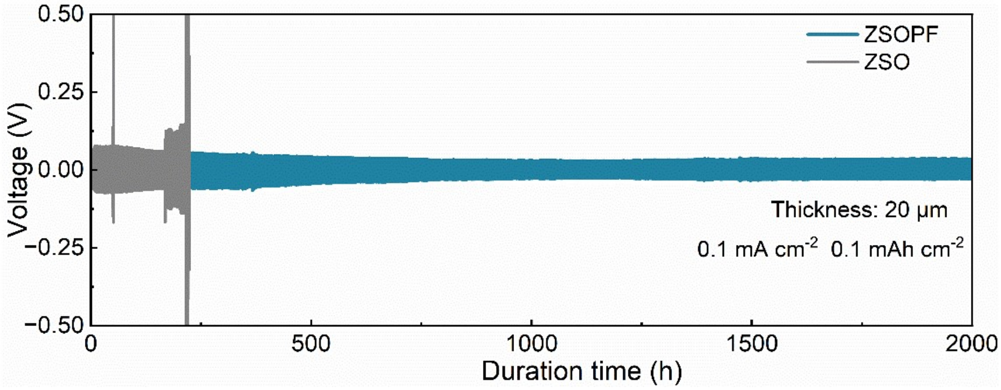

Fig. S16. Under the conditions of 0.1 mA cm^{-2} and 0.1 mAh cm^{-2}, the long-term constant current cycling of the Zn//Zn symmetric battery assembled with 20-μm-thick Zn foils in ZSO and ZSOPF electrolytes.

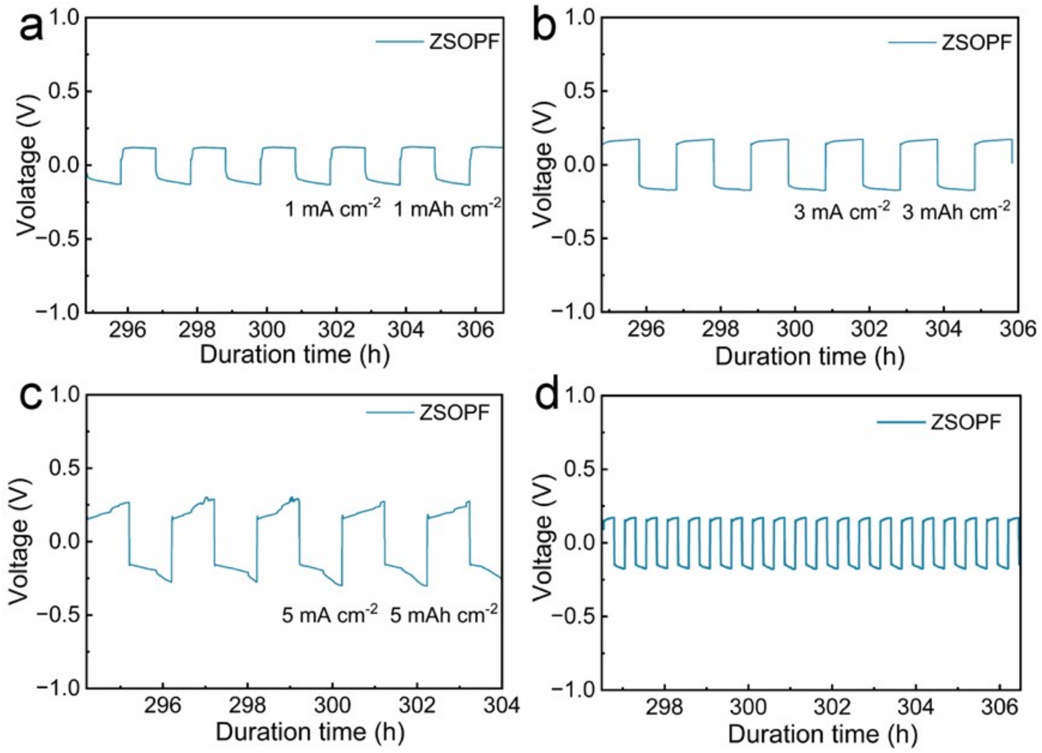

Fig. S17. Selected time-voltage magnified profiles of Zn//Zn symmetric batteries assembled with ZSOPF electrolyte at (a) 1 mA cm^{-2} and 1 mAh cm^{-2}, (b) 3 mA cm^{-2} and 3 mAh cm^{-2}, (c) 5 mA cm^{-2} and 5 mAh cm^{-2}, and (d) 5 mA cm^{-2} and 1.25 mAh cm^{-2}.

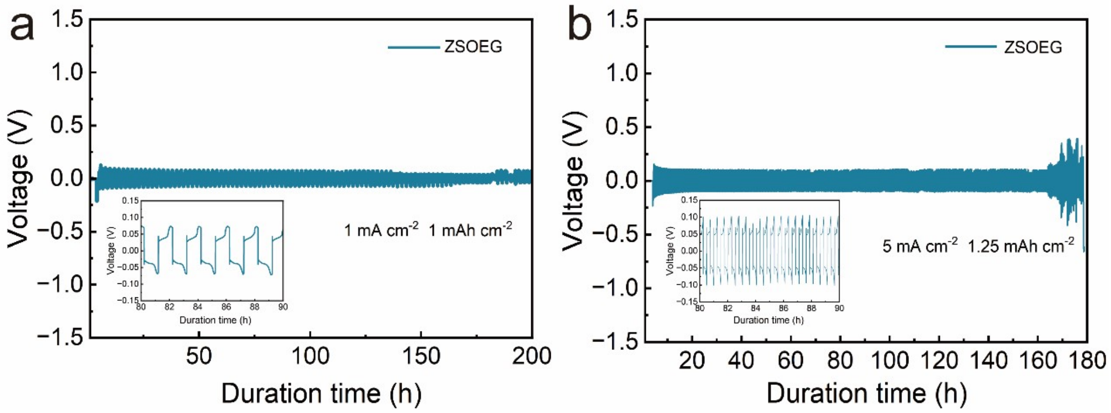

Fig. S18. Galvanostatic cycling of Zn//Zn symmetric batteries in ZSOEG at (a) 1 mA cm^{-2} and 1 mAh cm^{-2} and (b) 5 mA cm^{-2} and 1.25 mAh cm^{-2}.

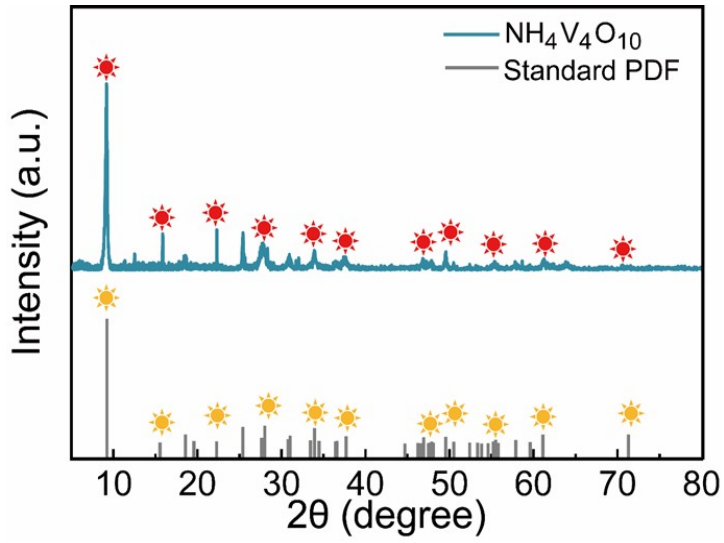

Fig. S19. XRD patterns of the hydrothermally synthesized $NH_4V_4O_{10}$ cathode compared with the standard $NH_4V_4O_{10}$ PDF card.

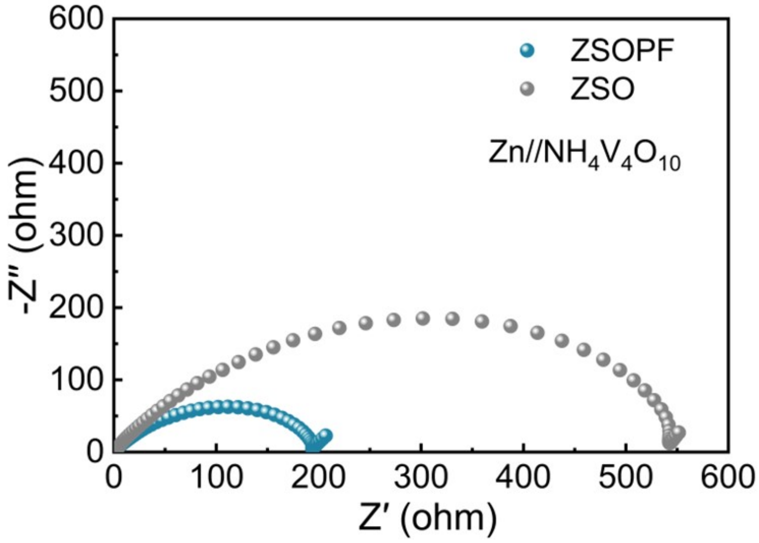

Fig. S20. The total impedance of the Zn//$NH_4V_4O_{10}$ battery in ZSO electrolyte and ZSOPF electrolyte.

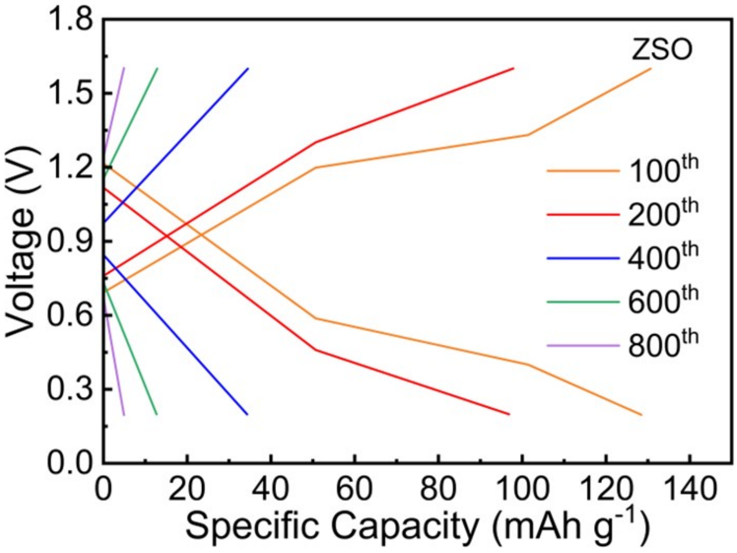

Fig. S21. Galvanostatic charge-discharge curves of Zn//$NH_4V_4O_{10}$ batteries at various cycle numbers under a current density of 5 A g^{-1}.

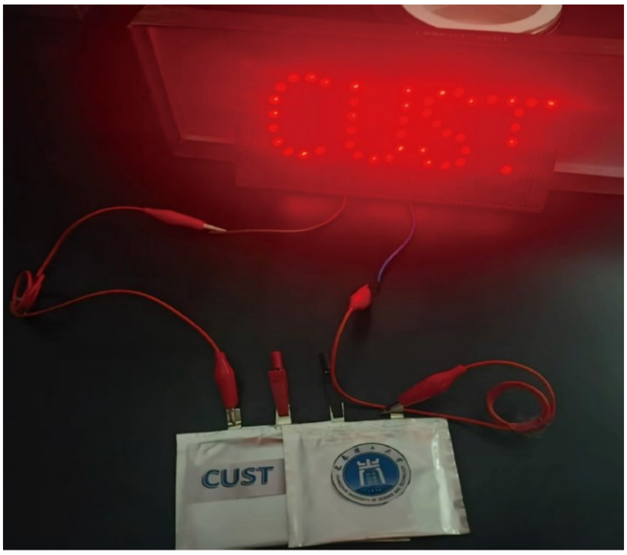

Fig. S22. Photograph of a flexible pouch cell stably powering a CUST-patterned LED light.

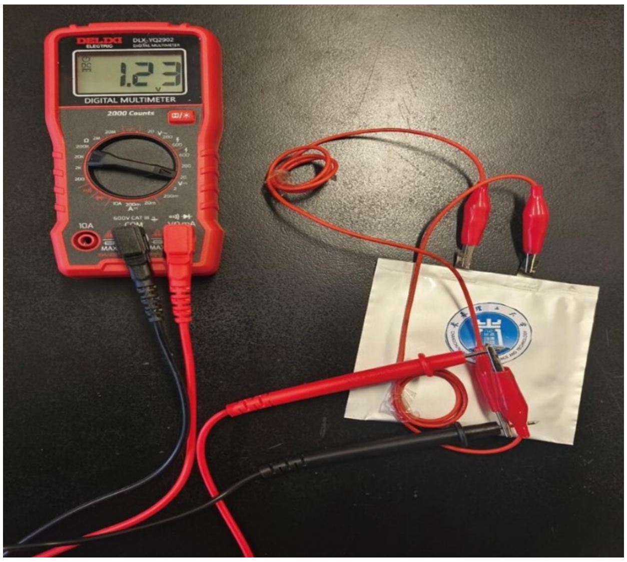

Fig. S23. Schematic diagram of the voltage of the pouch cell after stabilization.

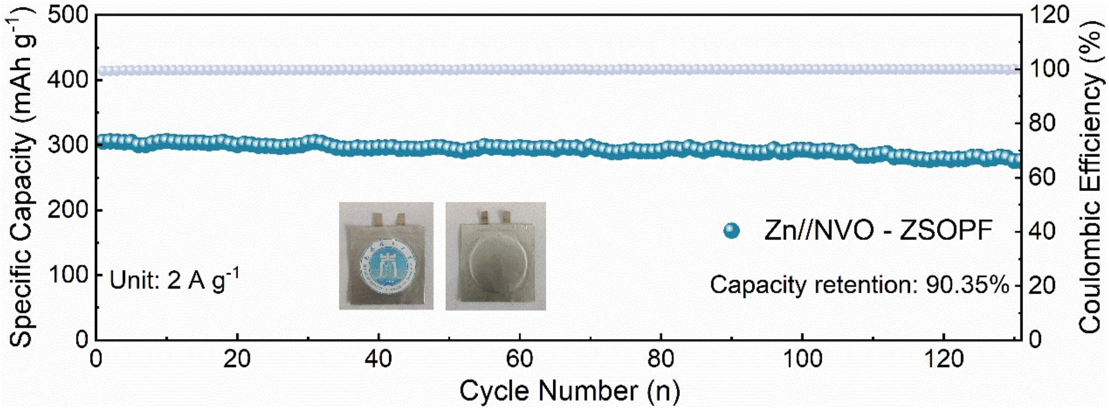

Fig. S24. Cycling performance of the pouch cell assembled with ZSOPF.

Notes and references

1. J. Zhang, Z. Liu, L. Jia, Y. Zhang, Q. Xie, W. Zhang, Y. Shi, B. Jiang and N. Zhang, *J. Colloid Interface Sci*., 2025, **686**, 613-623.
2. C. Wang, X. Wang, H. Wang, C. Zheng, S. Tan, Y. Wang, G. Diao and Z. Jin, *Adv. Funct. Mater.*, 2025, **35**, 2424024.
3. X. Hu, H. Dong, N. Gao, T. Wang, H. He, X. Gao, Y. Dai, Y. Liu, D. J. L. Brett, I. P. Parkin and G. He, *Nat. Commun*., 2025, **16**, 2316.
4. Q. Wang, X. Deng, X. Xue, J. Zhang, J. Zhao, Z. Sui, Y. Zou, L. Luo, W. Zhang, X. Liu and C. Lu, *Energy Environ. Sci.*, 2025, **18**, 5309-5318.
5. Z. Liu, G. Li, M. Xi, Y. Huang, H. Li, H. Jin, J. Ding, S. Zhang, C. Zhang and Z. Guo, *Angew. Chem. Int. Ed*., 2024, **63**, e202319091.
6. Y. Liu, L. Wu, P. Zhang, Y. Liu, J. Wu, S. Yao, L. Wang, S. Gong, G. Ju, Z. Yuan and R. Zhang, *J. Energy Chem*., 2024, **99**, 375-383.
7. W. Zhang, W. Qi, K. Yang, Y. Hu, F. Jiang, W. Liu, L. Du, Z. Yan and J. Sun, *Energy Storage Mater*., 2024, **71**, 103616.
8. X. Fan, L. Chen, Y. Wang, X. Xu, X. Jiao, P. Zhou, Y. Liu, Z. Song and J. Zhou, *Nano-micro Lett.*, 2024, **16**, 270.
